# Piezo1 Regulates Stiffness‐Dependent DRG Axon Regeneration via Modifying Cytoskeletal Dynamics

**DOI:** 10.1002/advs.202405705

**Published:** 2024-11-08

**Authors:** Mengshi Lei, Weiyou Wang, Hong Zhang, Jihong Gong, Hanmian Cai, Zhili Wang, Le Zhu, Xiaofei Yang, Shen Wang, Cong Ma

**Affiliations:** ^1^ Key Laboratory of Molecular Biophysics of the Ministry of Education College of Life Science and Technology Huazhong University of Science and Technology Wuhan 430074 China; ^2^ Key Laboratory of Cognitive Science Laboratory of Membrane Ion Channels and Medicine College of Biomedical Engineering South‐Central Minzu University Wuhan 430079 China; ^3^ Hubei Key Laboratory of Brain‐inspired Intelligent Systems Wuhan 430074 China

**Keywords:** axon regeneration, DRG neurons, Piezo1, substrate stiffness

## Abstract

Despite medical interventions, the regenerative capacity of the peripheral nervous system is limited. Dorsal root ganglion (DRG) neurons possess the capacity to detect mechanical signals from their microenvironment, but the impact and mechanism by which these signals regulate axon regrowth and even regeneration in DRG neurons remain unclear. In this study, DRG neurons from newborn rats are cultured on substrates with varying degrees of stiffness in vitro to investigate the role of mechanical signals in axon regrowth. The findings reveal that substrate stiffness plays a crucial role in regulating axon regrowth, with an optimal stiffness required for this process. In addition, the data demonstrate that Piezo1, a mechanosensitive cation channel, detects substrate stiffness at the growth cone and regulates axon regrowth through activating downstream Ca^2+^–CaMKII–FAK–actin cascade signaling pathway. Interestingly, knocking down Piezo1 in adult rat DRG neurons leads to enhanced axon regeneration and accelerated recovery of sensory function after sciatic nerve injury. Overall, these findings contribute to the understanding of the role of mechanical signals in axon regeneration and highlight microenvironmental stiffness as a promising therapeutic target for repairing nerve injuries.

## Graphical abstract

Despite medical interventions, the regenerative capacity of the peripheral nervous system remains limited. In this study, this challenge is addressed by identifying the Piezo1–Ca^2+^–CaMKII–FAK signaling cascade that regulates DRG axon regrowth in response to substrate stiffness by modulating actin retrograde flow. This coordinated signaling cascade presents a promising avenue for enhancing axon regeneration in vivo.

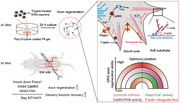

## 1 Introduction

In contrast to the central nervous system (CNS), the neurons in the peripheral nervous system (PNS) can regenerate their axons,^[^ ^1^, ^2^, ^3^ ^]^ despite limited regenerative capability.^[^ ^4^ ^]^ In some cases, severe peripheral nerve injuries can result in permanent neurological deficits, including chronic pain and abnormal perception. Elucidating the mechanism underlying axon regrowth following neuronal injury in the PNS and developing strategies to promote PNS axon regeneration is of significant importance in the treatment of PNS injuries and diseases.

Axon regrowth is a complex process in which the developing axon elongates and navigates the surrounding environment to reach its intended target. The growth cone, which is the leading edge of the axon, can sense and respond to external mechanical signals from the environment and control axon regrowth and regeneration by integrating these signals into internal cytoskeletal remodeling.^[^ ^5^, ^6^ ^]^ In the case of neuronal injury in DRG neurons, regenerated growth cones are subjected to altered mechanical force due to changes in extracellular matrix stiffness. For example, the formation of a glial scar can contribute to changes in the local microenvironment stiffness of DRG axons during axonal regeneration.^[^ ^7^, ^8^, ^9^ ^]^ Recent research has highlighted the influence of mechanical stress, tension, and extracellular matrix stiffness on axon regrowth and regeneration.^[^ ^10^, ^11^ ^]^ It has been found that membrane receptors, such as integrins, and mechanosensitive ion channels, such as Piezo1, TRPV2, and TRPC1, are involved in translating these mechanical signals into intracellular responses that affect axon regrowth.^[^ ^12^, ^13^, ^14^, ^15^ ^]^ Despite these findings, there have been relatively few comprehensive explorations of the impact of matrix stiffness on axon regrowth in DRG neurons.

It is known that Ca^2+^ plays a vital role in numerous cellular activities within neurons, including neurogenesis, axon growth, pathfinding, and regeneration.^[^ ^14^, ^16^, ^17^ ^]^ Previous studies have demonstrated that mechanosensitive cation channels, which allow the passage of Ca^2+^, can be activated by changes in matrix stiffness, consequently influencing Ca^2+^ activity.^[^ ^18^, ^19^ ^]^ In addition, Ca^2+^ activity has been found to regulate phosphorylation signaling cascades such as protein kinase C (PKC) and focal adhesion kinases (FAKs),^[^ ^20^ ^]^ which in turn influence the dynamics of the cytoskeleton.^[^ ^21^, ^22^ ^]^ Hence, it is intriguing to investigate whether and how stiffness‐mediated Ca^2+^ fluctuations regulate the process of axon regrowth and regeneration in DRG neurons.

In our study, we first conducted in vitro experiments where we cultured DRG neurons on substrates with different stiffness levels. Substrate stiffness‐dependent Ca^2+^ activity controls axon regrowth using a bidirectional regulative mechanism, where higher Ca^2+^ promotes axon retraction while lower Ca^2+^ facilitates axon extension, with an optimal Ca^2+^ required for efficient regrowth. Specifically, we identified the mechanosensitive cation channel Piezo1 as being sensitive to substrate stiffness, and it activates downstream Ca^2+^–CaMKII–FAK–actin cascade signaling pathway to orchestrate axon regrowth. Consistently, knocking down Piezo1 in adult rat DRG neurons leads to enhanced axon regeneration and accelerated recovery of sensory function after sciatic nerve injury. This study could shed light on the coordination of cellular responses during regeneration and potentially reveal new targets for therapeutic interventions aimed at promoting nerve regeneration.

## 2 Results

### 2.1 DRG Axon Regrowth Is Regulated by Substrate Stiffness In Vitro

**Figure 1:**
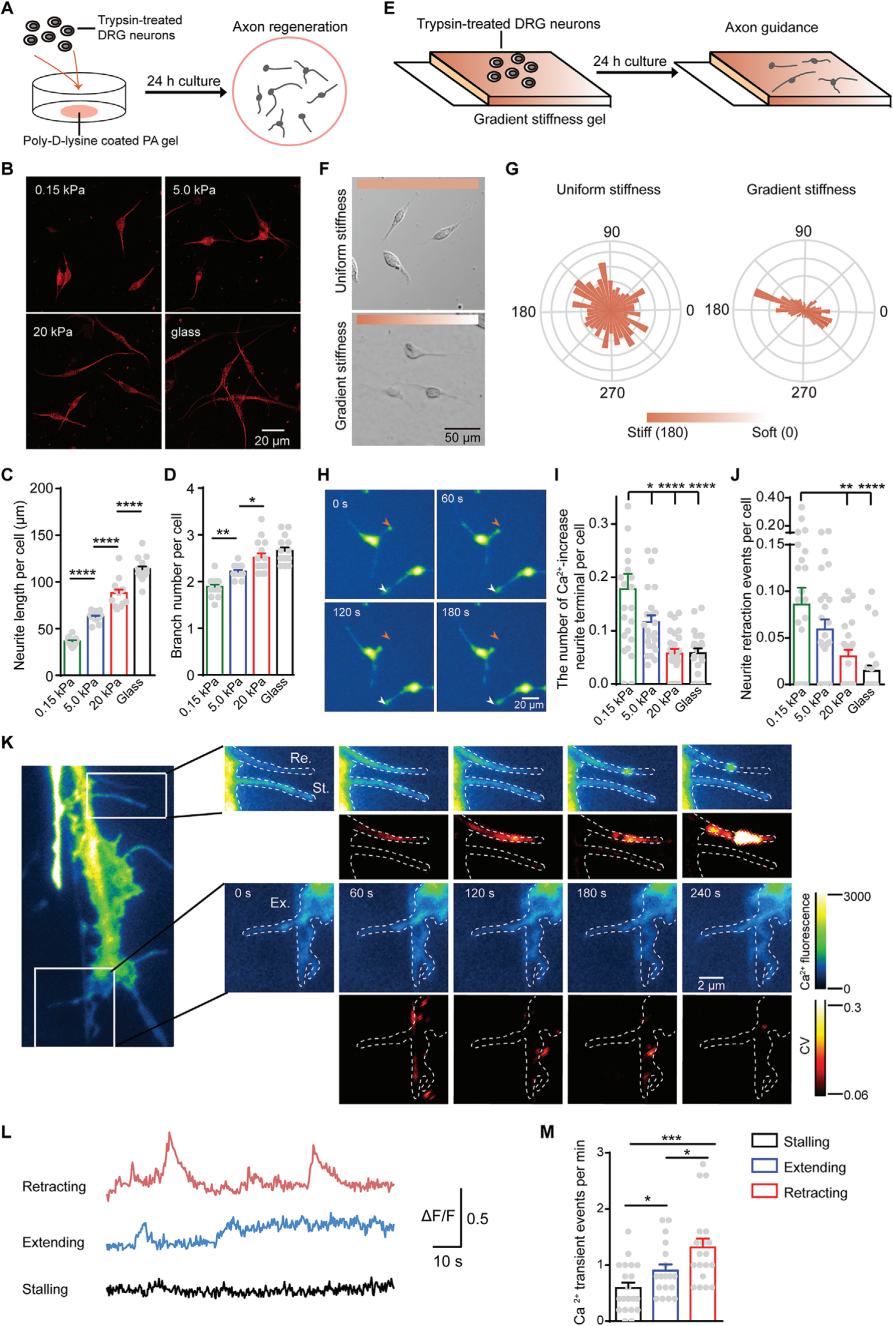
Substrate stiffness‐mediated Ca^2+^ activities modulates DRG axon regrowth. A) Experimental process of in vitro axon regeneration assay on different stiffness PA gels. B–D) Representative images (B) and quantification of neurite length (C) and branch number (D) per neuron cultured on 0.15, 5.0, and 20 kPa PA gels, and glass slides for 24 h. Each point on the panel represents the average total neurite length and branch number of a neuron per image containing 6–10 DRG neurons. E) Experimental process of in vitro axon guidance assay on gradient stiffness PA gels. F,G) Representative images (F) and angular displacement (G) of DRG axons on gradient stiffness PA gels for 1 day. 0° and 180° represent the soft side and stiff side, respectively. H) Representative real‐time calcium images of DRG neurons cultured on 0.15 kPa PA gels for 24 h, with neurite retraction events indicated by orange and white arrows. The arrows mark the initial positions of the retracted neurite terminals. I,J) Quantification of number of Ca^2+^‐increased neurite terminal (I) and neurite retraction events per neuron (J) cultured on 0.15, 5.0, and 20 kPa PA gels, and glass slides for 24 h. K) Representative real‐time calcium images of neurite terminal on glass slides for 1 day. The first and second‐row images on the right exhibit the retraction (Re.) terminal and static (St.) terminal, and the third and fourth‐row images exhibit the extension (Ex.) terminal. The first and third‐row images on the right display calcium fluorescence intensity; the second and fourth‐row images on the right display the CV of calcium intensity. The dotted lines indicate the initial morphology of neurite terminals. L,M) Representative traces of Ca^2+^ activity (L) and quantification of Ca^2+^ transient events per min (M) at neurite terminals undergoing retraction, extension, and stalling. Error bars denote mean ± SEM; *, *p* < 0.05; **, *p* < 0.01; ***, *p* < 0.001; ****, *p* < 0.0001, as determined by one‐way ANOVA (C–J) or two‐tailed Student's t‐test (M).

To investigate the impact of mechanical signal on axon regeneration in DRG neurons, we employed an in vitro axon regrowth assay (**Figure** **1**A) to replicate the process of in vivo axon regeneration following peripheral axotomy under various mechanical conditions.^[^ ^23^ ^]^ Specifically, we prepared polyacrylamide (PA) substrates with distinct elastic moduli of 0.15, 5.0, and 20 kPa to simulate different mechanical microenvironments. The 0.15 kPa approximated the lower range of brain tissue stiffness,^[^ ^24^ ^]^ while the 5.0 and 20 kPa were comparable to the physiological stiffness of DRGs.^[^ ^25^, ^26^ ^]^ Additionally, glass slides were used as the stiffest substrates due to their high stiffness (≈60 GPa). Then, trypsin‐treated DRG neurons from newborn rats were planted on different stiffness substrates coated with poly‐d‐lysine (Figure 1A).

We first assessed the DRG axon regrowth capability by characterizing the total neurite length and branch number of all branches per neuron. We observed a significant increase in both neurite length and branch number of DRG neurons with escalating substrate stiffness at day 1 in vitro (DIV1) (Figure 1B–D), indicating that mechanical cue from substrate stiffness plays a regulatory role in DRG axon elongation and branching. In addition, as successful axon regeneration requires appropriate axon guidance, we investigated the guidance of DRG axon in response to a gradient stiffness (4‒27 kPa, ≈3.0 kPa mm^−1^) (Figure 1E; Figure S1, Supporting Information). Compared to the random outgrowth of DRG neurites on the uniform stiffness substrate, DRG neurites on the gradient stiffness substrate exhibited clear directional growth along the stiffness gradient at DIV1 (Figure 1F,G), indicating that DRG axons can sense and respond to asymmetric mechanical signals from the gradient stiffness substrate to regulate axon guidance. While the role of asymmetric chemical signals in axon guidance is well documented,^[^ ^27^, ^28^ ^]^ our results unveil the importance of asymmetric mechanical signals in this process. Taken together, these results indicate that DRG axons are mechanosensitive and their regrowth is regulated by substrate stiffness.

### 2.2 Ca^2+^ Activity Determines Axon Regrowth

Considerable evidence supports the crucial role of cytosolic Ca^2+^ in axon growth and guidance.^[^ ^14^, ^29^ ^]^ As Ca^2+^ activity can be regulated by mechanical signals from substrate stiffness,^[^ ^30^, ^31^ ^]^ it is of interest to investigate the involvement of substrate stiffness‐dependent Ca^2+^ activity in DRG axon regrowth. To this aim, we performed calcium imaging using Fluo‐4 as a Ca^2+^ indicator on DRG neurons cultured on different stiffness substrates at DIV1 (Figure 1H). We observed a higher proportion of neurite terminals exhibiting Ca^2+^ influx on softer substrates (i.e., 0.15 and 5.0 kPa) compared to stiffer substrates (i.e., 20 kPa and glass) (Figure 1I), indicating that substrate stiffness modulates Ca^2+^ activity at DRG axon terminals.

Importantly, we observed the retraction phenomenon in regrowing neurite terminals of DRG neurons on different stiffness substrates, indicated by the withdrawal of neurite terminals towards the cell body. We found an increased frequency of neurite retraction events on softer substrates (Figure 1J), which were previously observed to have elevated Ca^2+^ activity (Figure 1H,I). Combined with the observed reduced axon regrowth on softer substrates (Figure 1B–D), we propose that Ca^2+^ activity influenced by substrate stiffness might regulate axon regrowth through mediating axon retraction.

To further validate the role of Ca^2+^ activity in influencing axon terminal regrowth, we conducted real‐time calcium imaging of neurite terminals on glass slides. Interestingly, we found neurite terminals displayed spontaneous local Ca^2+^ transients, which were associated with either retraction or extension (Figure 1K). We used the coefficient of variation (CV) as a measure of variability, with higher CV values indicating higher Ca^2+^ transients. Data showed that neurite terminals exhibiting a high CV value were more inclined to retract, while neurite terminals exhibiting a low CV value tended to extend (Figure 1K). Moreover, neurite terminals that rarely displayed Ca^2+^ transients tended to remain stationary (Figure 1K). In addition, an analysis of the Ca^2+^ transient frequency at neurite terminals revealed that retracting terminals exhibited a high frequency, whereas extending terminals displayed a low frequency (Figure 1L,M). Taken together, these results suggest that Ca^2+^ activity determines the fate of the neurite terminal's retraction or extension.

To examine the relationship between intracellular [Ca^2+^] and axon regrowth, we utilized ionomycin, a selective Ca^2+^ ionophore, and BAPTA‐AM, a permeable Ca^2+^ chelator, to manipulate intracellular Ca^2+^ levels. DRG neurons were cultured on glass slides and treated with different concentrations of ionomycin or BAPTA‐AM. After 24 h, we observed that DRG neurons treated with 1 µm ionomycin exhibited the maximum total neurite length and branch number of all branches compared to the control condition or treatments with higher concentrations of ionomycin (2, 5, or 10 µm) (Figure S2A,B, Supporting Information). Conversely, as the concentration of BAPTA‐AM increased, the neurite length and branch number decreased (Figure S2A,C, Supporting Information). These results suggest the presence of an optimal range of intracellular Ca^2+^ levels that promote optimal axon regrowth in DRG neurons.

### 2.3 Piezo1‐Mediated Ca^2+^ Influx Inhibits DRG Axon Regrowth

Mechanosensitive cation channels can sense substrate stiffness to mediate Ca^2+^ influx,^[^ ^12^, ^32^ ^]^ We thus investigated the potential role of mechanosensitive cation channels in the regulation of Ca^2+^‐mediated DRG axon regrowth in response to substrate stiffness. We initially treated DRG neurons cultured on various stiffness substrates with ruthenium red, a nonselective blocker that inhibits most mechanosensitive cation channels. Interestingly, compared to the control group without ruthenium red, we observed a significant increase in neurite length and branch number in DRG neurons cultured on softer substrates (i.e., 0.15 and 5.0 kPa), while this effect was not observed on stiffer substrates (i.e., 20 kPa and glass) (Figure S3, Supporting Information), suggesting the involvement of mechanosensitive cation channels in DRG axon regrowth.

**Figure 2:**
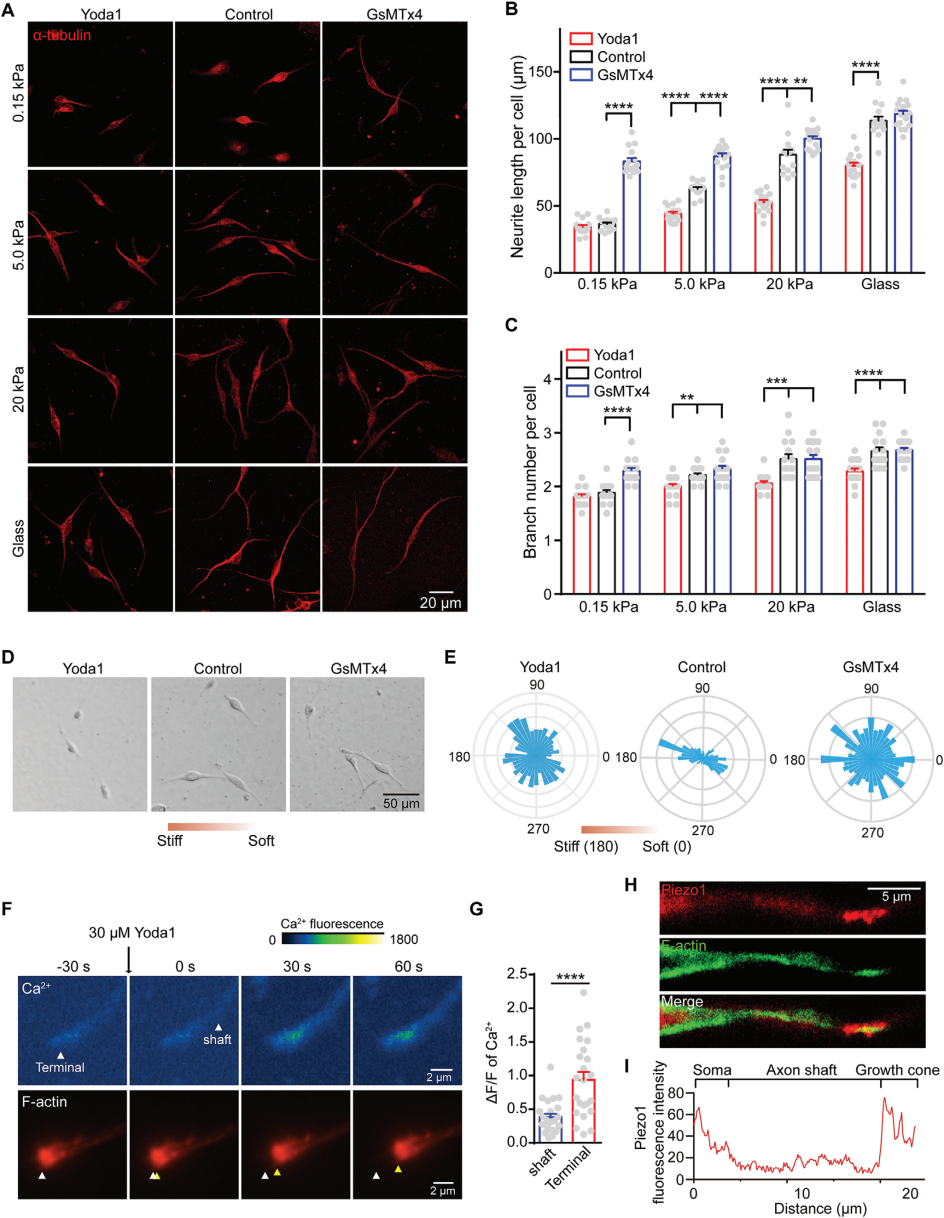
Piezo1‐mediated Ca^2+^ activities modulate stiffness‐dependent DRG axon regrowth. A–C) Representative images (A) and quantification of neurite length (B) and branch number (C) per neuron cultured on different substrate stiffness and incubated with media (control), 5 µm GsMTx4 or 30 µm Yoda1 for 24 h. D,E) Representative images (D) and angular displacement (E) of DRG axons cultured on gradient stiffness PA gels and incubated with media (control), 5 µm GsMTx4 or 30 µm Yoda1 for 24 h. F) Representative calcium (top) and F‐actin (bottom) images of DRG neurites cultured on glass slides for 24 h and stimulated with 30 µm Yoda1. The initial and real‐time positions of the neurite terminal are indicated by white and yellow arrows, respectively. G) Quantification of Yoda1‐induced calcium *ΔF/F* at neurite shaft and terminal as ascribed in panel F). *ΔF/F*, (*F*_Yoda1_‐*F*_basal_)/F_basal_, basal (*F*_basal_), and Yoda1 stimulated fluorescence intensity (*F*_Yoda1_) of neurite was calculated. H,I) Representative immunofluorescence images of Piezo1 and F‐actin (H) and Piezo1 fluorescence intensity along the direction of neuron soma‐axon shaft‐growth cone (I). Error bars denote mean ± SEM; **, *p* < 0.01; ***, *p* < 0.001; ****, *p* < 0.0001, as determined by two‐tailed Student's t‐test.

TRPV1 and Piezo1 mediate Ca^2+^ influx in response to mechanical signals and participate in various biological processes.^[^ ^18^, ^33^ ^]^ TRPV1 and Piezo1 are expressed in DRG neurons.^[^ ^34^, ^35^, ^36^ ^]^ To evaluate the potential role of TRPV1, we utilized capsazepine, a specific inhibitor of this channel. We observed a significant suppression of DRG axon regrowth on all tested stiffness substrates (Figure S3, Supporting Information), indicating that TRPV1 regulates axon regrowth independent of substrate stiffness. In contrast, when we inhibited Piezo1 using its inhibitor GsMTx4, we observed a significant enhancement of DRG axon regrowth on relatively soft substrates, with no observed effect on stiff substrates (**Figure** **2**A–C). Conversely, the selective agonist of Piezo1, Yoda1, notably inhibited axon regrowth on relatively stiff substrates, while showing no such effect on soft substrates (Figure 2A–C). The stronger inhibitory effect of GsMTx4 on axon regrowth on softer substrates suggests that Piezo1 was activated by soft substrates, consistent with our previous study in DRG neurons.^[^ ^37^ ^]^ In addition, we found that the addition of GsMTx4 or Yoda1 abolished guidance of DRG axons on gradient stiffness substrate in comparison with the control group (Figure 2D,E), suggesting that the manipulation of Piezo1 eliminates its asymmetric activation induced by gradient stiffness. In summary, these findings suggest that Piezo1 mediates DRG axon regrowth by sensing substrate stiffness.

To further probe the function of Ca^2+^ mediated by Pieoz1 in regulating axon regrowth, we monitored the Ca^2+^ transients and morphological dynamics of DRG axons stimulated with Yoda1. Our data showed that Yoda1 treatment resulted in a significantly greater Ca^2+^ influx at axon terminals compared to the axon shaft (Figure 2F,G), implying that Piezo1 is enriched at axon terminals. In addition, we observed a noticeable retraction of neurite terminals following Yoda1 stimulation (Figure 2F), suggesting a correlation between retraction and Ca^2+^ influx mediated by Piezo1. Furthermore, immunofluorescence imaging of Piezo1 demonstrated its co‐localization with F‐actin in the growth cone (Figure 2H,I), similar to our previous observations (Figure 2F,G). Taken together, our data suggest that Piezo1 functions through Ca^2+^ signaling to regulate axon regrowth in response to substrate stiffness.

### 2.4 CaMKII Functions Downstream of Piezo1 to Regulate DRG Axon Regrowth

We then investigated the downstream pathway through which Piezo1 functions in DRG axon regrowth via Ca^2+^ signaling in response to substrate stiffness. We focused on Ca^2+^/calmodulin‐dependent protein kinase II (CaMKII), which has been reported to be activated by Piezo1‐mediated Ca^2+^ influx.^[^ ^14^, ^38^ ^]^ Additionally, it has been established that CaMKII plays a significant role in axon development and regeneration.^[^ ^39^, ^40^ ^]^ Thus, it is of interest to investigate whether CaMKII functions as a downstream effector of Piezo1 in regulating axon regrowth.

**Figure 3:**
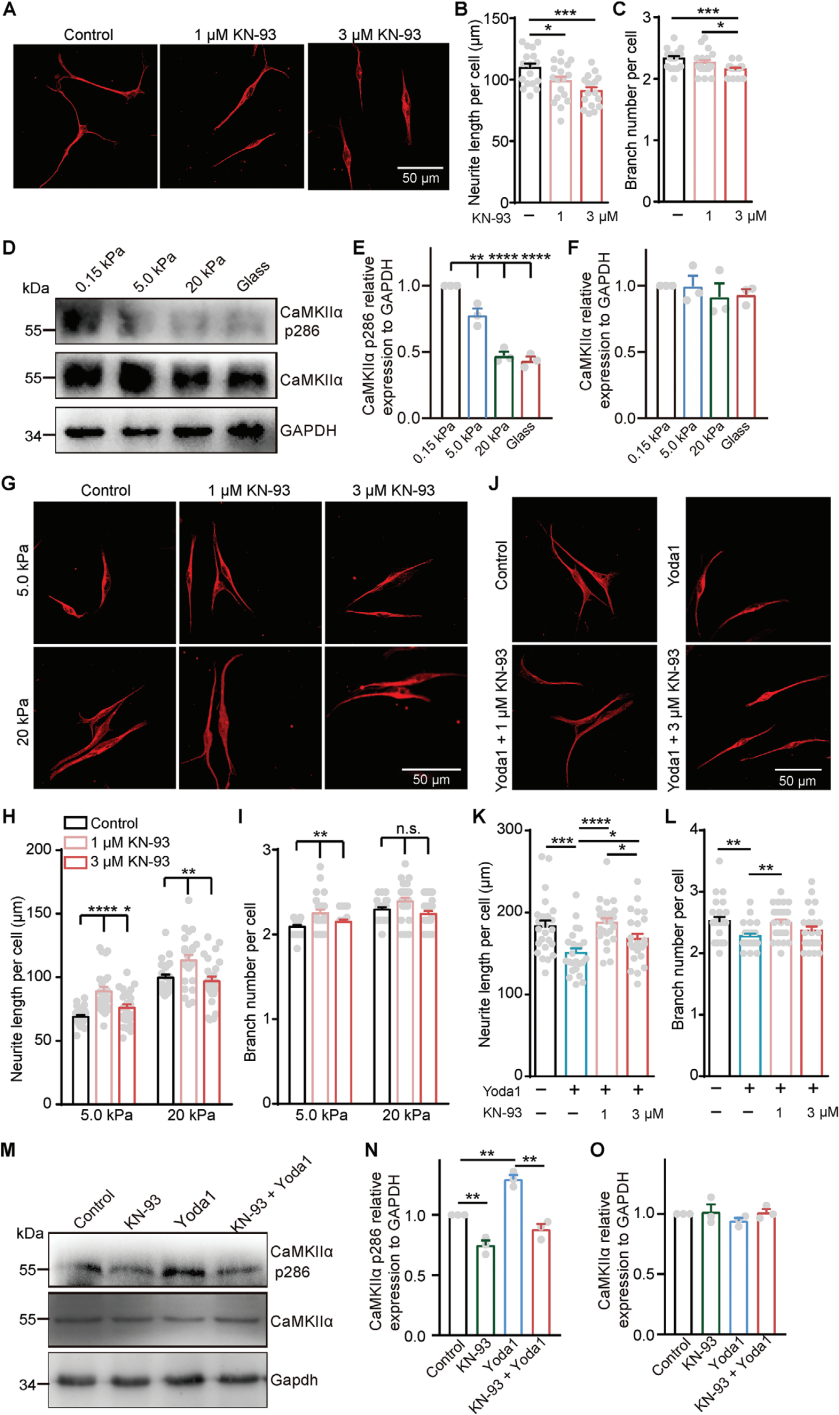
CaMKII serves as a downstream effector of stiffness and Piezo1 regulates DRG axon regrowth. A–C) Representative images (A) and quantification of neurite length (B) and branch number (C) per neuron cultured on glass slides and treated with media (control), 1 µm KN‐93 or 3 µm KN‐93 for 24 h. D–F) Western blots (D) and quantification of CaMKIIα p286 (E), CaMKIIα (F) in whole cell lysis of DRG neurons seeded on different stiffness substrates, GAPDH used as the reference protein. G–I) Representative images (G) and quantification of neurite length (H) and branch number (I) per neuron cultured on 5.0 and 20 kPa PA gels and treated with media (control), 1 µm KN‐93 or 3 µm KN‐93 for 24 h. J–L) Representative images (J) and quantification of neurite length (K) and branch number (L) per neuron cultured on glass slides and treated with media (control), 30 µm Yoda1 and/or 1 µm KN‐93, 3 µm KN‐93 for 24 h. M–O) Western blots (M) and quantification of CaMKIIα p286 (N), CaMKIIα (O) in whole cell lysis of DRG neurons seeded on glass slides and treated with media (control), 3 µm KN‐93 and/or 30 µm Yoda1 for 24 h, GAPDH used as the reference protein. Error bars denote mean ± SEM; *, *p* < 0.05; **, *p* < 0.01; ***, *p* < 0.001; ****, *p* < 0.0001, as determined by one‐way ANOVA (E–F) or two‐tailed Student's t‐test (B, C, and H–O).

To evaluate the contribution of CaMKII in DRG axon regrowth, we treated DRG neurons cultured on glass slides with KN‐93, a specific cell‐permeable inhibitor of CaMKII. Our findings demonstrated a significant reduction in neurite length and branch number in DRG neurons treated with either 1 µm or 3 µm KN‐93 compared to the control group without KN‐93 (**Figure** **3**A–C), indicating that the activity of CaMKII plays an essential role in axon regrowth. Next, we investigated whether CaMKII is regulated by substrate stiffness and influences DRG axon regrowth. Western blot analysis revealed a decrease in the phosphorylation level of CaMKII at Thr286 (p‐CaMKII^T286^) with increasing substrate stiffness, while the expression level of CaMKII remained unaffected (Figure 3D–F). Since the p‐CaMKII^T286^ is indicative of CaMKII activity,^[^ ^41^ ^]^ this finding suggests that the activity of CaMKII is indeed influenced by substrate stiffness. Furthermore, after knocking down CaMKII using lentivirus, we observed a significant influence of CaMKII on stiffness‐mediated DRG axon regrowth (Figure S4A–C, Supporting Information). Specifically, we found that axon regrowth was enhanced in CaMKII knockdown neurons on soft substrates (i.e., 0.15 and 5 kPa), while it was inhibited on glass slides (Figure S4A–C, Supporting Information). To delve deeper into the regulatory role of CaMKII in stiffness‐mediated axon regrowth, we cultured DRG neurons on soft substrates (i.e., 5.0 or 20 kPa) and treated them with varying concentrations of KN‐93 (1 and 3 µm) to modulate CaMKII activity to different extents. Surprisingly, compared to the control group without KN‐93, the neurite length was significantly increased following treatment with 1 µm KN‐93 (29.0% on 5.0 kPa vs 13.8% on 20 kPa) or 3 µm KN‐93 (10.2% on 5.0 kPa versus −3.7% on 20 kPa) (Figure 3G–I). The effect of KN‐93 on DRG axon regrowth on the soft substrate was more pronounced than those on the stiff substrate, indicating that CaMKII exhibits increased activity with reduced stiffness.

Then, we investigated whether CaMKII exerts an effect on Piezo1‐mediated DRG axon regrowth. Intriguingly, mild inhibition of CaMKII with low concentration (1 µm) of KN‐93 eliminated the inhibitory effect of Yoda1 on axon regrowth in DRG neurons cultured on glass slides (Figure 3J–L), indicating that CaMKII may serve as a downstream effector of Piezo1 in axon regrowth. In addition, robust inhibition with high concentration (3 µm) of KN‐93 exhibited a less pronounced effect in reversing the decreased axon regrowth induced by Yoda1 compared to 1 µm KN‐93, implying the existence of an optimal range of CaMKII activity that facilitates axon regrowth. To further confirm this observation, we applied varying concentrations of KN‐93 to DRG neurons cultured on glass slides with or without Yoda1 treatment. Without Yoda1 treatment, we found that increasing the concentration of KN‐93 significantly decreased the neurite length and branch number of DRG neurons (Figure S4D,E, Supporting Information). Additionally, in the presence of 30 µm Yoda1, axon regrowth was notably enhanced by lower concentrations of KN‐93, while higher concentrations of KN‐93 had no significant effect (Figure S4D,F, Supporting Information). These results suggest that the effects of KN‐93 on axon regrowth are dose‐dependent, indicating the presence of an optimal activity of CaMKII for effective DRG axon regrowth.

We also investigated the impact of Piezo1 on the phosphorylation level of CaMKII at Thr286. Immunoblotting analysis of CaMKII and p‐CaMKII^T286^ in DRG neurons cultured on glass slides revealed that treatment with Yoda1 significantly increased the level of p‐CaMKII^T286^, while treatment with KN‐93 reduced the phosphorylation level of CaMKII^T286^ (Figure 3M–O). Importantly, KN‐93 eliminated the Yoda1‐induced increase in p‐CaMKII^T286^, indicating that CaMKII acts downstream of Piezo1 in axon regrowth. It is noteworthy that the expression level of CaMKII was not affected by either Yoda1 or KN‐93 treatment (Figure 3M–O). These findings provide further evidence for the involvement of CaMKII as a downstream effector of Piezo1 in axon regrowth.

### 2.5 FAK Functions Downstream of Piezo1‐CaMKII to Regulate DRG Axon Regrowth

**Figure 4:**
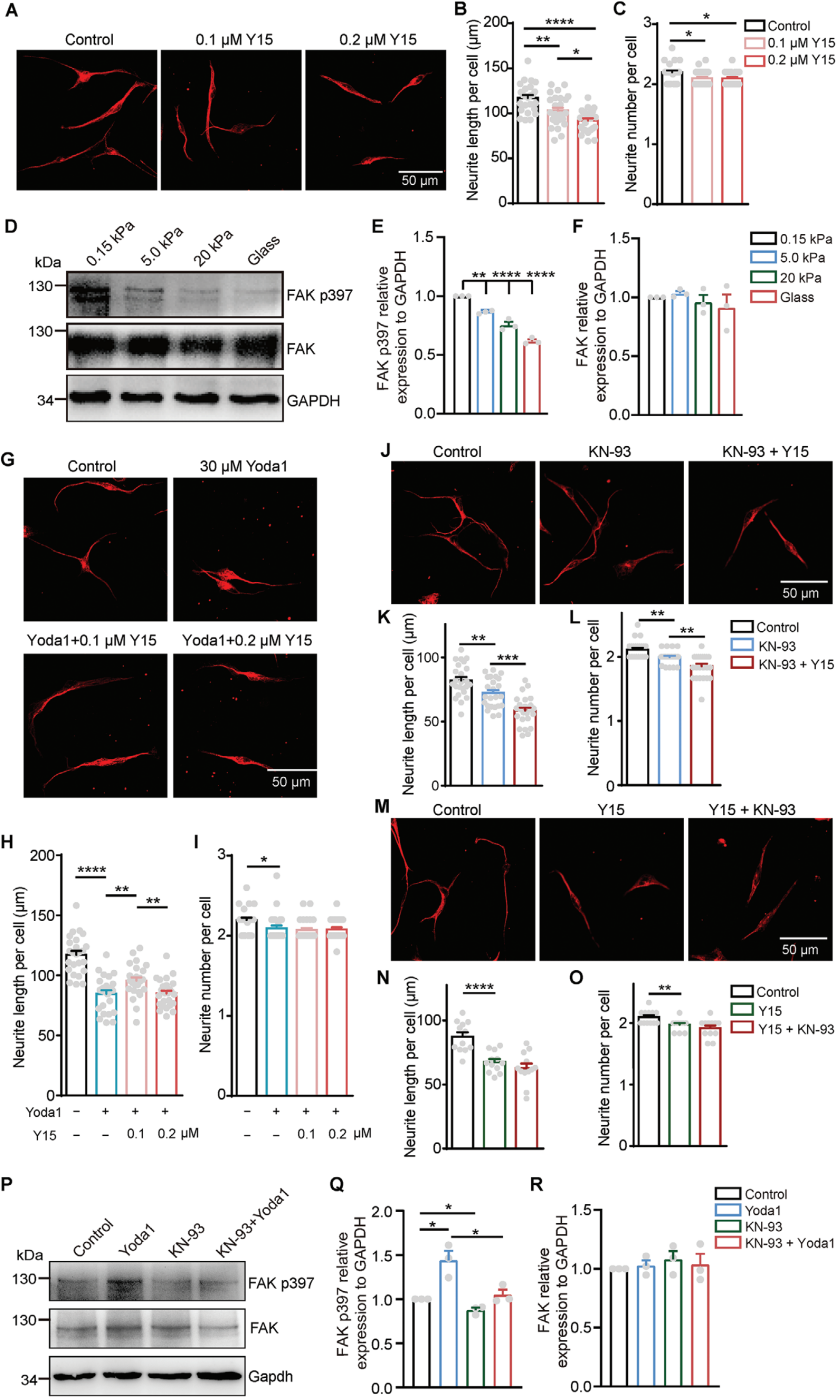
FAK acts as a downstream effector of Piezo1 and CaMKII to regulate DRG axon regrowth. A–C) Representative images (A) and quantification of neurite length (B) and branch number (C) per neuron cultured on glass slides and treated with media (control), 0.1 µm Y15 or 0.2 µm Y15 for 24 h. D–F) Western blots (D) and quantification of FAK p397 (E), FAK (F) in whole cell lysis of DRG neurons seeded on different stiffness substrates, GAPDH used as the reference protein. G–I) Representative images (G) and quantification of neurite length (H) and branch number (I) per neuron cultured on glass slides and treated with media (control), 30 µm Yoda1 and/or 0.1 µm Y15, 0.2 µm Y15 for 24 h. J–L) Representative images (J) and quantification of neurite length (K) and branch number (L) per neuron cultured on glass slides and treated with media (control), 3 µm KN‐93, and/or 0.3 µm Y15 for 24 h. M–O) Representative images (M) and quantification of neurite length (N) and branch number (O) per neuron cultured on glass slides and treated with media (control), 0.3 µm Y15 and/or 3 µm KN‐93 for 24 h. P–R) Western blots (P) and quantification of FAK p397 (Q), FAK (R) in whole cell lysis of DRG neurons seeded on glass slides and treated with media (control), 30 µm Yoda1 and/or 3 µm KN‐93 for 24 h, GAPDH used as the reference protein. Error bars denote mean ± SEM; *, *p* < 0.05; **, *p* < 0.01; ***, *p* < 0.001; ****, *p* < 0.0001, as determined by one‐way ANOVA (E–F) or two‐tailed Student's t‐test (B, C and H–R).

Previous studies have shown that focal adhesion kinase (FAK) can be activated by Ca^2+^ signaling and plays a crucial role in mechanotransduction.^[^ ^42^, ^43^ ^]^ To assess the role of FAK in axon regrowth, we used Y15, a specific inhibitor that targets the autophosphorylation of FAK at Tyr397, subsequently inhibiting FAK activity.^[^ ^44^, ^45^ ^]^ Our data showed that treatment with 0.1 µm or 0.2 µm Y15 resulted in a decrease in neurite length and branch number of DRG neurons cultured on glass slides compared to control group without Y15 (**Figure** **4**A–C), indicating that FAK plays a significant role in DRG axon regrowth. Next, we investigated whether FAK is regulated by substrate stiffness and influences DRG axon regrowth. Western blot analysis revealed a decrease in the phosphorylation level of FAK at Tyr397 with increasing substrate stiffness, while the expression level of FAK was unaffected (Figure 4D–F). Since the p‐FAK^Y397^ reflects the FAK activity, this finding suggests that substrate stiffness indeed influences FAK activity. Furthermore, upon knocking down FAK using lentivirus, we observed a notable influence of FAK on stiffness‐mediated DRG axon regrowth (Figure S5A–C, Supporting Information). Specifically, axon regrowth was enhanced in FAK knockdown neurons on soft substrates (i.e., 0.15 kPa), while it was inhibited on glass slides (Figure S5A–C, Supporting Information). These findings suggest that FAK plays an important role in axon regrowth mediated by substrate stiffness.

We then examined whether FAK was involved in Piezo1‐mediated DRG axon regrowth. Interestingly, treatment with 30 µm Yoda1 inhibited axon regrowth, and mild inhibition of FAK with low concentration (0.1 µm) of Y15 partially reversed this suppression (Figure 4G–I), indicating FAK acts as a downstream effector of Piezo1 in DRG axon regrowth. Moreover, robust inhibition of FAK with high concentration (0.2 µm) of Y15 has no effect on the Yoda1‐induced suppression of axon regrowth (Figure 4G–I). These results suggest that FAK functions downstream of Piezo1 in a dose‐dependent manner.

To further confirm these findings, we conducted a dose‐response experiment by treating DRG neurons cultured on glass slides with various concentrations of Y15, with or without Yoda1. Under the condition without Yoda1, we observed a significant decrease in neurite length and branch number of DRG neurons as the concentration of Y15 increased (Figure S5D,E, Supporting Information). In addition, in the presence of 30 µm Yoda1, lower concentrations of Y15 significantly enhanced axon regrowth, whereas higher concentrations of Y15 actually inhibited axon regrowth (Figure S5D,F, Supporting Information). These results demonstrate that Y15 modulates axon regrowth in a dose‐dependent manner, similar to the pattern observed with KN‐93, suggesting the presence of an optimal activity of FAK for effective DRG axon regrowth.

Considering the role of CaMKII as a downstream effector of Piezo1 (Figure 3), we sought to investigate the interplay between CaMKII and FAK in axon regrowth. Interestingly, the combination of Y15 with KN‐93 exhibited an intensified inhibitory effect on axon regrowth compared to DRG neurons treated only with KN‐93 (Figure 4J–L), while it had no impact on axon regrowth compared to DRG neurons treated with Y15 alone (Figure 4M–O). These results suggest that FAK acts as a downstream effector of CaMKII in DRG axon regrowth.

Furthermore, we performed immunoblotting analysis to assess the phosphorylation level of FAK at Tyr397 (p‐FAK^Y397^) in DRG neurons cultured on glass slides. We observed that Yoda1 treatment led to an increase in the level of p‐FAK^Y397^, signifying the activation of FAK by Yoda1 (Figure 4P–R). Importantly, this enhancement in p‐FAK^Y397^ was abolished by KN‐93, suggesting that FAK serves as a downstream effector of both Piezo1 and CaMKII (Figure 4P–R). Collectively, these findings suggest that FAK is activated by Piezo1 and Piezo1‐CaMKII‐FAK signaling pathway functions in DRG axon regrowth.

### 2.6 Piezo1 and Ca^2+^ Regulate Actin Dynamics in the Growth Cone to Mediate Axon Regrowth

**Figure 5:**
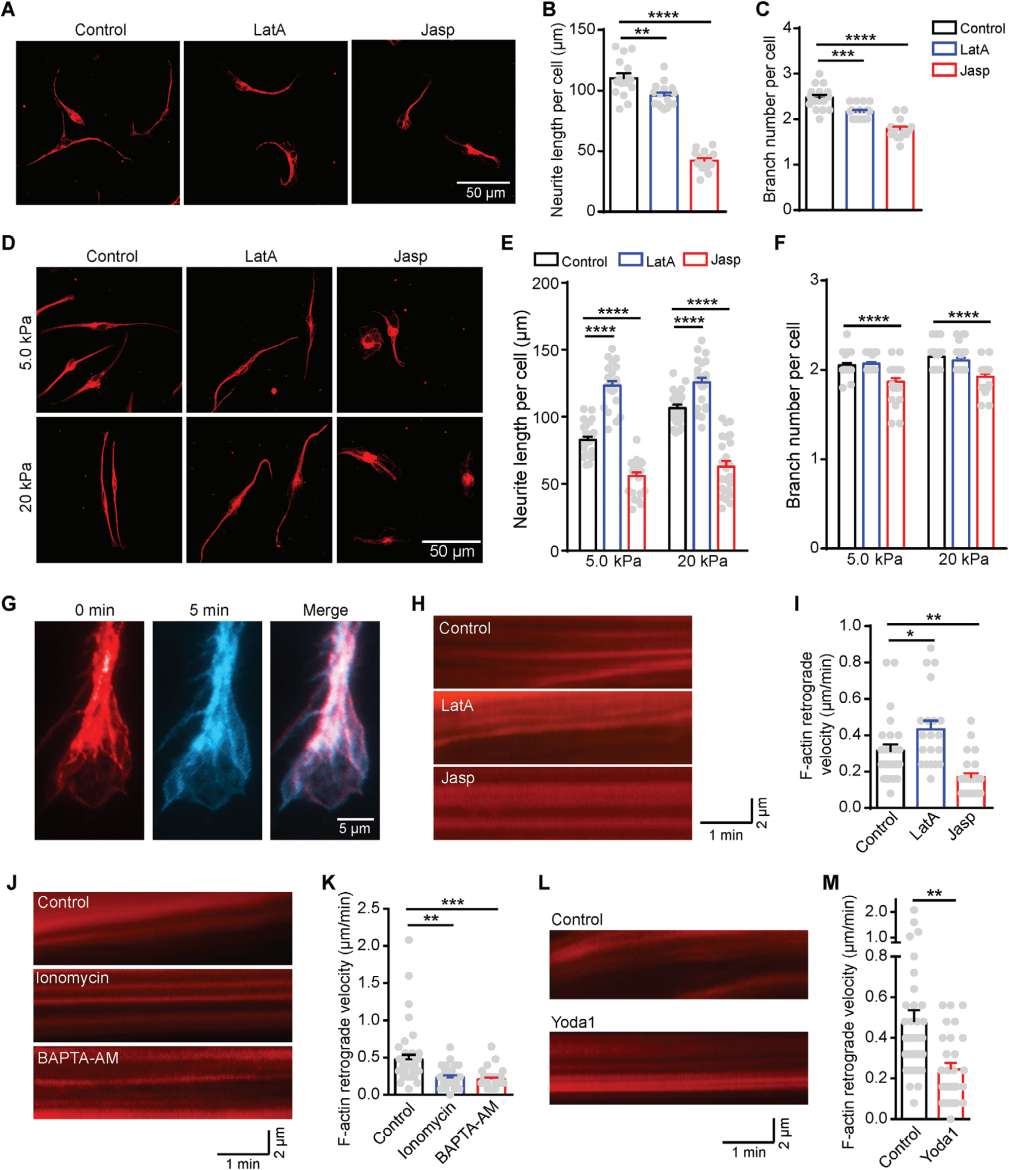
F‐actin retrograde flow controls DRG axon regrowth. A–C) Representative images (A) and quantification of neurite length (B) and branch number (C) per neuron cultured on glass slides and treated with media (control), 0.1 µm LatA or 0.1 µm Jasp for 24 h. D–F) Representative images (D) and quantification of neurite length (E) and branch number (F) per neuron cultured on 5.0 and 20 kPa PA gels and treated with media (control), 0.1 µm LatA or 0.1 µm Jasp for 24 h. G) Representative images of F‐actin in growth cone showing the dynamics of actin over a period of 5 min. H,I) Representative images (H) and quantification of F‐actin retrograde flow velocity (I) in growth cone of DRG cultured on glass slides and treated with media (control), 0.5 µm LatA, or 0.5 µm Jasp before recording. J,K) Representative images (J) and quantification of F‐actin retrograde flow velocity (K) in growth cone of DRG cultured on glass slides and treated with media (control), 5 µm Ionomycin, or 1 µm BAPTA‐AM before recording. L,M) Representative images (L) and quantification of F‐actin retrograde flow velocity (M) in the growth cone of DRG cultured on glass slides and treated with media (control) or 30 µm Yoda1 before recording. Error bars denote mean ± SEM; *, *p* < 0.05; **, *p* < 0.01; ***, *p* < 0.001; ****, *p* < 0.0001, as determined by two‐tailed Student's t‐test.

Previous studies have demonstrated that external mechanical signaling can alter cytoskeleton dynamics, a pivotal factor in regulating axon regeneration.^[^ ^5^, ^46^ ^]^ Here, our investigation revealed the regulatory role of FAK in controlling F‐actin thickness beneath the plasma membrane (Figure S6A,B, Supporting Information) and actin dynamics (Figure S6C,D, Supporting Information). Based on these observations, we hypothesize that actin acts as a downstream effector of Piezo1, directly influencing axon regrowth. To test this hypothesis, we manipulated actin dynamics in DRG neurons using the actin polymerization inhibitor latrunculinA (LatA) and the actin polymerization inducer jasplakinolide (Jasp). Both LatA and Jasp inhibited axon regrowth on glass slides (**Figure** **5**A–C). Notably, LatA promoted axon regrowth on relatively soft substrates (i.e., 5.0 and 20 kPa), while Jasp exhibited an inhibitory effect (Figure 5D–F). These results provide compelling evidence that actin dynamics are affected by substrate stiffness and play a regulatory role in DRG axon regrowth.

Some evidence has demonstrated that actin retrograde flow, associated with actin dynamics, plays a role in regulating axon regeneration.^[^ ^47^, ^48^ ^]^ Thus, we quantified the velocity of actin retrograde flow in growth cones of newborn DRG neurons to investigate the impact of actin dynamics on axon regrowth (Figure 5G). LatA and Jasp were utilized to modulate the F‐actin retrograde flow by influencing actin turnover.^[^ ^49^ ^]^ Remarkably, LatA treatment increased the rate of actin retrograde flow, while Jasp decreased it (Figure 5H,I). These results are consistent with our findings on soft substrates (i.e., 5.0 and 20 kPa) (Figure 5D–F), suggesting that promoting actin retrograde flow with LatA enhances DRG axon regrowth, while Jasp treatment, decreasing F‐actin retrograde flow, inhibits DRG axon regrowth. However, both LatA and Jasp inhibit DRG axon regrowth on stiff substrates like glass (Figure 5A–C). It could be due to that LatA initially increases actin retrograde flow within a short duration (10 min), while prolonged exposure (24 h) to excessive retrograde flow of actin may disrupt the balance of actin dynamics and inhibit axon regrowth. Furthermore, LatA and Jasp might regulate DRG axon regrowth through mechanisms other than F‐actin retrograde flow.

Subsequently, we examined whether Piezo1 and Ca^2+^ are involved in the regulation of actin retrograde flow to influence axon regrowth. Our data revealed that manipulating Ca^2+^ levels using ionomycin or BAPTA‐AM significantly decreased the retrograde flow of actin in the growth cone (Figure 5J,K), indicating that Ca^2+^ signaling can modulate actin dynamics. Furthermore, Yoda1, known to inhibit axon regrowth through activating Piezo1‐mediated Ca^2+^ influx, significantly suppressed actin retrograde flow (Figure 5L,M). These findings demonstrate that Piezo1 regulates actin dynamics in the growth cone through Ca^2+^ signaling, altering the actin retrograde flow, and ultimately governing axon regrowth. Collectively, these results provide evidence that the Piezo1–CaMKII–FAK–actin signaling cascade is responsible for regulating stiffness‐mediated DRG axon regrowth.

### 2.7 Piezo1–CaMKII–FAK Regulates DRG Axon Regeneration and Function Recovery After Injury

The identification of an optimal condition for Piezo1–CaMKII–FAK signaling pathway in axon regrowth led us to hypothesize that there exists an optimal range of substrate stiffness required for Piezo1 activity to promote DRG axon regeneration. In light of our previous results, we propose that the optimal substrate stiffness falls within the range of 20 kPa to glass. Due to the limited stiffness of PA hydrogels, we utilized PDMS as a substrate material to achieve higher stiffness (2, 50, 100, 200, and 300 kPa). Surprisingly, we observed that DRG axon regrowth was the most effective on 200 kPa substrate (Figure S7, Supporting Information). This optimal stiffness creates an ideal environment for the activation of Piezo1–CaMKII–FAK–actin signaling cascade, ultimately facilitating axon regrowth.

**Figure 6:**
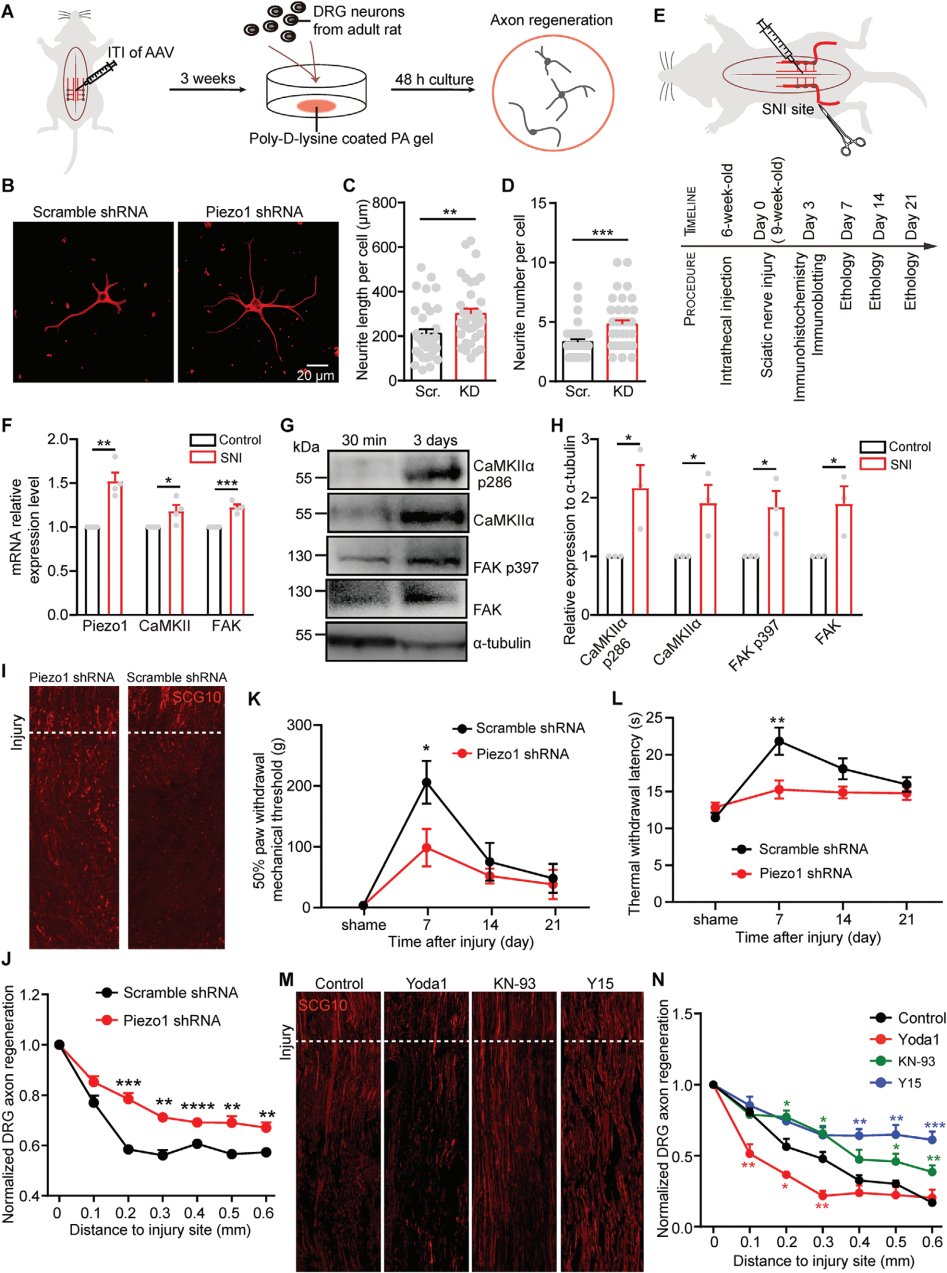
Piezo1‐CaMKII‐FAK signaling cascade regulates repair after axon injury in vivo. A) Experimental process of intrathecal injection (ITI) virus and in vitro axon regeneration assay performed in adult rats. B–D) Representative images (B) and quantification of neurite length (C) and branch number (D) per neuron infected with control (Scramble shRNA) or Piezo1 knockdown (Piezo1 shRNA) virus and cultured on 5.0 kPa PA gels for 48 h. E) Experimental process of SNI and ethology assay. F) Relative *Piezo1*, *CaMKIIα*, *Fak* mRNA expression of DRG in control (without injury) or day 3 after SNI rats, *Gapdh* used as reference gene. G,H) Western blots (G) and quantification (H) of CaMKIIα p286 and CaMKIIα, FAK p397, and FAK at the SNI site with 3 days post‐injury or 30 min post‐injury, α‐tubulin used as the reference protein. I,J) Representative immunofluorescence images of SCG10 at longitudinal sections (I) and quantification of relative SCG10 intensity plotted in function of the distance from the injury site (J) in control and Piezo1 knockdown DRGs. The injury site is indicated by dotted line, *n* = 4 for each column. K,L) Summary graphs of rat 50% paw withdrawal mechanical threshold (K) and thermal withdrawal latency (L) of DRGs infected with scramble or Piezo1 shRNA. Each point in (K and L) denotes experiments from an individual rat, *n* = 12 for each column. M,N) Representative immunofluorescence images of SCG10 at longitudinal sections (M) and quantification of relative SCG10 intensity plotted in function of the distance from the injury site (N) in control (corn oil), 300 µm Yoda1, 10 µm KN‐93 or 10 µm Y15 treatment DRGs. The injury site is indicated by a dotted line, *n* = 4 for each column. Error bars denote mean ± SEM; *, *p* < 0.05; **, *p* < 0.01; ***, *p* < 0.001; ****, *p* < 0.0001, as determined by two‐tailed Student's t‐test.

The significant role of Piezo1 in mediating in vitro axon regrowth of DRG prompted us to investigate its function in axon regeneration after injury in vivo. To evaluate this, we employed intrathecal injection of Piezo1 shRNA into 6‐week‐old rats to knock down Piezo1 in DRGs (L3‐L6) (Figure S8, Supporting Information). Three weeks post‐injection, we cultured trypsin‐treated DRG neurons from adult rats on a soft substrate (5.0 kPa) coated with poly‐D‐lysine and assessed their axon regeneration capability at DIV2 (**Figure** **6**A). Remarkably, our observation reinforces that Piezo1 knockdown in DRG neurons enhanced axon regeneration on relatively soft substrates (Figure 6B–D).

We next investigate the function of Piezo1 in in vivo DRG axon regeneration under physiological conditions with several kPa stiffness. We performed sciatic nerve injury (SNI) to damage DRG axons and assess the axon regeneration capacity from both morphology and ethology perspectives (Figure 6E). Interestingly, on day 3 post‐SNI, qPCR analysis revealed an upregulation of Piezo1, CaMKII, and FAK at the injured side compared to the uninjured side (Figure 6F). Moreover, Western Blot analysis showed that expression levels of CaMKII and p‐CaMKII^T286^, FAK, and p‐FAK^Y397^ at the injury site were elevated on day 3 post‐SNI compared to levels observed 30 min after injury (Figure 6G,H). These findings suggest that Piezo1, CaMKII, and FAK play an important role in regulating axon repair after injury. Next, we utilized SCG10, a marker of regenerating sensory axons, to identify the regenerative axon of DRG neurons at day 3 post‐SNI. Surprisingly, we found the extension SCG10^+^ axon was significantly increased in Piezo1‐deficient DRGs compared to the control group (scramble shRNA) (Figure 6I,J), indicating that Piezo1 suppresses axon regeneration.

We also conducted behavior tests including thermal and mechanical perception to assess the degree of sensory function recovery following SNI (Figure 6E). For the mechanical response, we applied a set of von Frey hair filaments to measure the paw‐withdrawal mechanical threshold. At day 7 post‐SNI, rats with Piezo1 knockdown in DRGs showed significantly increased responses to mechanical stimulus compared to the control rats, as indicated by a decreased paw‐withdrawal mechanical threshold (Figure 6K). For the thermal response, we recorded the paw‐withdrawal latency of rats in response to thermal radiation. Consistent with the mechanical response finding, rats with Piezo1 knockdown in DRGs exhibited significantly increased responses to thermal stimulus compared to the control rats, as indicated by a decreased paw‐withdrawal latency (Figure 6L). Moreover, both the control and Piezo1 knockdown rats displayed similar thermal and mechanical responses starting from day 14 post‐SNI (Figure 6K,L). Taken together, these findings suggest that Piezo1 inhibits in vivo axon regeneration and consequently hinders the recovery of sensory function in adult DRG neurons.

To further investigate the role of the Piezo1–CaMKII–FAK signaling cascade in axon regeneration in vivo, we manipulated the activity of Piezo1, CaMKII, and FAK at the injured site in adult rats that underwent SNI. We applied Yoda1, KN‐93, or Y15 to modulate the activity of Piezo1, CaMKII, and FAK, respectively, and used SCG10 to identify the regenerative axon at day 3 post‐SNI. Compared to the control group treated with corn oil, Yoda1 substantially suppressed axon regeneration (Figure 6M,N). Conversely, KN‐93 or Y15 treatment exhibited a more effective axon regenerative (Figure 6M,N). These results are consistent with our in vitro findings, suggesting that Piezo1, CaMKII, and FAK are excessively activated under physiological conditions and that we can promote axon repair after injury by suppressing the Piezo1–CaMKII–FAK signaling pathway.

## 3 Discussion

**Figure 7:**
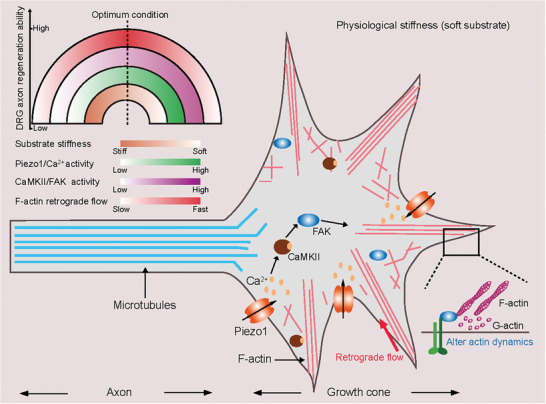
Piezo1‐CaMKII‐FAK‐actin signaling cascade regulates DRG axon regeneration in the growth cone. A proposed model for the substrate stiffness‐mediated DRG axon regrowth/regeneration through Piezo1‐CaMKII‐FAK‐actin signaling cascade. The diagram in the top left depicts the relationship between substrate stiffness (orange), Piezo1/ Ca^2+^ activity (green), CaMKII/FAK activity (purple), F‐actin retrograde flow velocity (red), and DRG axon regeneration ability. We suggest that achieving an optimum condition of substrate stiffness, Piezo1/ Ca^2+^ activity and CaMKII/FAK activity is essential for optimal F‐actin retrograde flow velocity and ultimately promoting optimal axon regrowth/regeneration.

PNS axons have the inherent capacity to regenerate after injury, but the recovery is typically slow and limited, even when nerve grafts are employed to assist with regeneration.^[^ ^1^, ^50^ ^]^ A considerable number of researches have implicated the involvement of mechanical signals in DRG axon regeneration. However, we still have a limited understanding of the underlying mechanism by which matrix stiffness regulates axon regeneration. In this study, we address this question and identify the Piezo1–Ca^2+^–CaMKII–FAK signaling cascade that regulates DRG axon regrowth in response to substrate stiffness by modulating actin retrograde flow. This coordinated signaling cascade holds promise as a potential avenue for enhancing axon regeneration in vivo.(**Figure** **7**)

All cells are situated within mechanical microenvironments, which encompass the stiffness of the extracellular matrix or neighboring cells. The mechanical microenvironments play a vital role in regulating various physiological processes, including morphogenesis, cell migration, and histogenesis.^[^ ^24^, ^51^, ^52^ ^]^ Consistent with precious studies that have elucidated substrate stiffness as a critical signal in influencing CNS axon regrowth and pathfinding,^[^ ^53^, ^54^ ^]^ our findings highlight the importance of substrate stiffness in regulating the regrowth of DRG axons. Specifically, we observe that DRG neurons cultured on a substrate with a stiffness of 200 kPa, which exceeds physiological stiffness, exhibited the highest regenerative capacity. This discovery has important implications for the development of nerve guidance conduits (NGCs),^[^ ^55^, ^56^ ^]^ as it offers the potential to engineer NGCs with optimal stiffness to effectively promote PNS axon regeneration. Besides, based on our findings indicating that a soft substrate suppresses DRG axon regeneration, we propose that the loss of regenerative ability in central nervous system (CNS) axons may be attributed to the soft physiological stiffness of the brain (0.1–1 kPa).

A previous study has already indicated the involvement of Piezo1 in DRG axon regeneration,^[^ ^10^, ^12^ ^]^ and our study serves to validate and provide a deeper understanding of the mechanisms at play in this process. Our findings reveal that Piezo1 is enriched in the growth cone and is activated by soft substrates surrounding DRG neurons. This might arise because soft substrates promote focal adhesion dynamics,^[^ ^37^ ^]^ thus activating Piezo1 by creating changes in membrane curvature.^[^ ^57^ ^]^ In addition, our results are consistent with our earlier study demonstrating that Piezo1 is activated by soft substrates in the soma of DRG neurons.^[^ ^37^ ^]^ Our in vitro study utilized substrates with stiffness closer to physiological conditions, which advances our knowledge of the function of Piezo1 in the in vivo axon regeneration after injury. As a result, we propose that Piezo1 is directly activated by soft substrates to inhibit axon regeneration.

In vivo, the knockdown of Piezo1 in DRGs eliminates the inhibitory effect of soft physiological stiffness on axon regeneration, leading to an accelerated recovery of sensory perception following axon injury. Consistent with previous reports indicating the enrichment of Piezo1 in the growth cone and its regulation of axon regeneration after axon injury in *Drosophila*,^[^ ^14^ ^]^ we observe that Piezo1 is enriched in the growth cone of DRG neurons and up‐regulated after sciatic nerve injury in rats, elucidating that Piezo1 plays a role in axon regeneration in a spatiotemporally coherent manner. Based on our in vivo and in vitro findings indicating that inhibiting or knocking down Piezo1 in DRG neurons promotes axon regrowth/regeneration, we suggest that Piezo1 holds promise as a novel therapeutic target for treating axon injuries. This finding could have significant implications for the development of targeted therapies aimed at promoting axon regeneration following nerve injuries.

The function of Piezo1 primarily depends on the activation‐induced Ca^2+^ influx.^[^ ^58^, ^59^ ^]^ Our findings establish that the local Ca^2+^ activities in the growth cone control DRG axon regrowth. The role of Ca^2+^ in axon regeneration has been debated, with conflicting views regarding whether it promotes or inhibits axon regeneration.^[^ ^60^, ^61^ ^]^ Our study contributes to resolving this long‐standing debate by demonstrating the bidirectional role of Ca^2+^ in this process, where higher Ca^2+^ promotes axon retraction while lower Ca^2+^ facilitates axon extension. Specifically, both excessive and insufficient levels of Ca^2+^ inhibit DRG axon regeneration, while an optimal Ca^2+^ concentration leads to the best DRG axon regeneration. Our results are consistent with prior research in endothelial tip cells, confirming the critical role of Ca^2+^ activity within the growth cone in determining the fate of branch elongation or retraction.^[^ ^62^ ^]^ Additionally, we note that other Ca^2+^ channels, such as TRPV1 located in the growth cone of DRG axons, can regulate axon regeneration irrespective of substrate stiffness, suggesting that multiple channels modulating Ca^2+^ activity in the growth cone regulate axon regeneration. Although Piezo1 is not the only mediator of local Ca^2+^ transients associated with axon regrowth, we declare that substrate stiffness‐dependent axon regrowth is mainly attributed to Piezo1.

The downstream effectors of Piezo1 in the signaling pathway for regulating axon regeneration have been elucidated in our research, revealing that CaMKII and FAK serve as crucial components. Our study demonstrates the dual role of CaMKII and FAK in axon regeneration, indicating their potential for both positive and negative regulation based on differences in activity levels. This highlights the existence of optimal activity levels for promoting axon regeneration, adding to our understanding of the intricate regulatory mechanisms involved in this process. Furthermore, recent studies have emphasized the critical role of cytoskeletal dynamics in axon regeneration.^[^ ^5^, ^6^ ^]^ In our research, we observe that Piezo1, Ca^2+^, or FAK have the potential to alter the actin retrograde flow, with faster retrograde flow correlating with a better axon regeneration capacity. Additionally, activating Piezo1 at the injury site suppresses axon regeneration after SNI, while inhibiting CaMKII and FAK promotes it. Therefore, our study provides a promising therapeutic avenue for axon injury by manipulating the Piezo1–CaMKII–FAK–actin pathway.

This comprehensive understanding of the signaling pathway involved in substrate stiffness‐mediated DRG axon regrowth, including its associated effectors and their impact on cytoskeletal dynamics and axon regeneration, offers valuable insights for developing targeted therapies aimed at promoting axon regeneration following nerve injuries. Manipulating the stiffness of NGCs and Piezo1–CaMKII–FAK–actin pathway holds significant potential as a novel approach for treating axon injuries, offering new avenues for therapeutic intervention in this critical area of neurological research.

## 4 Experimental Section

### Antibodies and Reagents

The following antibodies were used for immunolabelling: Rabbit IgG (Proteintech, rabbit polyclonal, 30000‐0‐AP), anti‐Piezo1 (Proteintech, rabbit polyclonal, 15939‐1‐Ig), anti‐βIII‐Tubulin (Abclonal, mouse monoclonal, A18132), anti‐GAPDH (Proteintech, mouse monoclonal, 60004‐1‐Ig), anti‐Phospho‐FAK (Tyr397) (Cell signaling technology, rabbit clonal, 3283S), anti‐Phospho‐CaMKII (Tyr286) (Abclonal, rabbit polyclonal, AP0255), anti‐CaMKII (Abclonal, rabbit polyclonal, A2508), anti‐FAK (Abclonal, rabbit polyclonal, A11195). The following reagents were used: Yoda1 (Sigma, SML1558), GsMTx4 (Abcam, ab141871), BAPTA‐AM (Yeasen, 50404ES25), Ionomycin (Yeasen, 50401ES03), Capsazepine (MedChemExpress, HY15640), Ruthenium red (MedChemExpress, HY‐103311), KN‐93 (MedChemExpress, HY‐15465A), Y15 (MedChemExpress, HY‐12444), Fluo‐4 (Invitrogen, F14201), Latrunculin (APExBIO, B7555), Jasplakinolide (Santa Cruz, sc‐202191), CellMask Orange Actin Tracking Stain (Invitrogen, A57244), FITC Phalloidin (Yeasen, 40735ES75).

### DRG Neurons Culture

DRGs from Sprague‐Dawley rat pups or adult rats were dissociated with 0.25% Trypsin‐EDTA (Gibco) and 0.1% collagenase II (Sigma) for 40 or 120 min, respectively. The dissociated DRG neurons were then planted in DMEM medium (Gibco) with 10% FBS (Gibco) for a duration of 4 h. Afterward, the medium was replaced with Neurobasal complete medium (Gibco) supplemented with 2% B27 (Gibco), 200 µm L‐glutamine, and 1% Penicillin‐Streptomycin (Gibco) to support the cell growth. The neurons were maintained in a cell incubator (Thermo) at a temperature of 37 °C and a CO_2_ concentration of 5%. After cultured 24 h, the total length and number of all branches per neuron were calculated to assess axon regrowth capacity.

All animal procedures strictly adhered to the animal use guidelines of Huazhong University of Science and Technology and received the necessary approvals from the relevant animal use committees.

### Preparation of Polyacrylamide (PA) Gels

Polyacrylamide (PA) gels with different elasticity were fabricated as previously described.^[^ ^63^, ^64^ ^]^ Cover‐glass‐bottom dishes (Cell E&G) were the first to be pre‐treated for polyacrylamide (PA) gels production. The dishes underwent an overnight treatment with 0.1 M NaOH (Vetec) and then a 10‐min incubation with N‐[3‐(Trimethoxysilyl) propyl] ethylenediamine. After being washed three times, the dishes were treated with 0.5% glutaraldehyde for one h. PA gels were polymerized on the cover‐glass‐bottom dishes using a 15 µL mixture comprised of 40% w/v acrylamide (Vetec), 2% w/v N,N‐methylene‐bis‐acrylamide (Biosharp), 0.05% w/v ammonium persulfate (BBI Life Science) and 0.05‰ N,N,N9,N9‐ tetramethylethylenediamine (TEMED; Bio‐Rad). To ensure stable polymerization of PA‐gels on the glass bottom of the dishes, a 12 mm diameter round glass was attached on the top. The homogeneous (constant Young's modulus) gels (0.15, 5.0, and 20 kPa PA gels) were produced by combining specific amounts of acrylamide and bis‐acrylamide as previously described. In brief, the 0.15 kPa PA gel consisted of 3% acrylamide and 0.04% N,N‐methylene‐bis‐acrylamide. The 5.0 kPa PA gel was composed of 5% acrylamide and 0.15% N,N‐methylene‐bis‐acrylamide. The 20 kPa PA gel consisted of 12% acrylamide and 0.2% N,N‐methylene‐bis‐acrylamide.

Before use, PA gels were exposed to UV irradiation of Sulfo‐SNAPAH (CovaChem) in a solution of 100 mm HEPES at pH 8.2 for functionalization. Finally, the gels were coated with 0.1 mg mL^−1^ poly‐D‐lysine (Sigma) and incubated at 4 °C overnight.

### Fabrication of Stiffness Gradient Polyacrylamide (PA) Hydrogel

Stiffness gradient hydrogel was fabricated using a two‐step polymerization process as previously described.^[^ ^65^ ^]^ Briefly, aliquots (250 µL) containing 9% acrylamide monomer (Vetec) and 0.4% N, N methylene‐bis‐acrylamide (Biosharp) cross‐linker were poured into a glass mold treated with dichlorodimethylsilane (DCDMS). A glass coverslip, treated with 3‐(trimethoxysilyl)‐propyl methacrylate (3‐TMPM), was then placed on top of the mold, resulting in the polymerization chamber taking the shape of a right‐angled ramp with a 3° angle in the vertical plane. After the first polymerization step and removal from the mold, a second 280‐µL aliquot of 9% acrylamide and 0.4% N, N methylene‐bis‐acrylamide was poured onto the first gel. Upon the polymerization of the second component, the compound structure is removed from the mold, yielding a stiffness gradient hydrogel composed of two sequentially polymerized components.

Before use, PA gels were exposed to UV irradiation of Sulfo‐SNAPAH (CovaChem) in a solution of 100 mm HEPES at pH 8.2 to be functionalized. Finally, the gels were coated with 0.1 mg mL^−1^ poly‐D‐lysine (Sigma) and incubated at 4 °C overnight.

### Ca^2+^ Imaging

Simultaneous monitoring of the Ca^2+^ activity and morphology of DRG neurons was carried out on neurons cultured in vitro at DIV1. Neurons were loaded with 1 mm Fluo‐4 AM (Invitrogen) by incubating them in freshly prepared buffer A at 37 °C for 40 min. Buffer A contained 20 mm HEPES, 1 mm MgSO4, 3.3 mm Na2CO3, 1.3 mm CaCl2, 0.1% BSA (w/v), and 2.5 mm Probenecid in HBSS (Gibco) at pH 7.4. Excess Fluo‐4 was removed by washing the neurons with buffer A. To visualize Fluo‐4 fluorescence, the dye was excited using a wavelength of 488 nm, detected with an EMCCD (Electron Multiplying Charge Coupled Device, ANDOR, iXon3). The camera captured frames at a rate of 10 Hz for a total duration of 300 seconds. The acquired fluorescent signals were then analyzed using NIS‐Elements AR 4.40.00 software from Nikon.

### Western Blotting

Culturing cells on glass slides were placed on ice and rinsed twice with ice‐cold phosphate‐buffered saline (PBS). The cells were scraped into an SDS lysis buffer containing 50 mm Tris‐HCl (pH 6.8), 100 mm DTT, 2.0% sodium dodecyl sulfate (SDS), and 10% glycerol. The lysates were collected, sonicated, and subsequently heated at 100 °C for 15 min. Next, the lysates were separated by SDS–polyacrylamide gel electrophoresis and then transferred to nitrocellulose membranes. The membranes were blocked with 5% bovine serum albumin (BSA) for 1 h and incubated with the primary antibodies for two h at room temperature, followed by fluorophore‐conjugated secondary antibodies for 1–2 h at room temperature. All the antibodies were diluted in the PBST buffer (137 mm NaCl, 2.7 mm KCl, 10 mm Na_2_HPO_4_, 2 mm NaH_2_PO_4_, and 0.5% Tween‐20). Finally, the membranes were scanned using a FluorChem FC3 imaging system (ProteinSimple).

### Immunofluorescent Staining

DRG neurons cultured at DIV1 were fixed in 4% paraformaldehyde for 10 min and permeabilized with 0.2% Triton X‐100 for 8 min. Following PBS washed the cells were blocked in 5% BSA for 30 min prior to being incubated with primary antibodies for 2 h at room temperature or overnight at 4 °C. The cells were then repeatedly washed with PBS and incubated with secondary antibodies which were diluted in 5% BSA, for 2 h at room temperature. The secondary antibodies used were Alexa Fluor‐488 goat anti‐mouse (Invitrogen) and Alexa Fluor‐546 goat anti‐rabbit (Invitrogen) antibodies.

Images were captured using a Nikon C2 confocal microscope that was equipped with a 60 × oil‐immersion or 20 × objective lens. Identical settings were applied to all samples within each experiment.

### Actin dynamics Imaging

For the analysis of actin dynamics in the growth cone, the CellMask orange actin tracking stain (Invitrogen) was utilized. DRG neurons cultured in vitro at DIV1 were loaded with 1 mm CellMask in the medium by incubating them at 37 °C for 50 min. Excess dye was removed by washing the neurons with PBS. The dye was excited using a wavelength of 532 nm, and TIRF (Total Internal Reflection Fluorescence microscopy) was applied to track the F‐actin at the growth cone. For the quantification of actin retrograde flow speed, kymographs were performed using the Fiji KymoResliceWide plugin.

### Stable Downregulation of CaMKII, FAK, and Piezo1 in DRG Neurons

The AAV virus was used to stably downregulate CaMKIIα,^[^ ^66^ ^]^ FAK,^[^ ^67^ ^]^ and Piezo1 in DRG neurons in vivo, the shRNA sequences were as follows:

Non‐target (scramble) shRNA,

(CCTAAGGTTAAGTCGCCCTCGTTCAAGAGACGAGGGCGACTTAACCTTAGG);

Piezo1 shRNA,

(CACCGCGTCTTTCTCAGCCACTACTTTCAAGAGAAGTAGTGGCTGAGAAAGACGC);

CaMKIIα shRNA,

(CCACTACCTTATCTTCGAT*TTCAAGAGA*ATCGAAGATAAGGTAGTGG);

FAK shRNA,

(GGTCCAGACCAATCACTATTTCAAGAGAATAGTGATTGGTCTGGACC).

### RNA Extraction and Real‐Time Fluorescence Quantification PCR (RT‐qPCR)

Total RNA was extracted from lumbar (L)3/L4/L5 DRG tissue using TRIzol RNA isolation following the manufacturer's protocols. The purity of total RNA ranged between 1.9 and 2.0. The purified total RNA was converted into complementary DNA (cDNA) utilizing the reverse‐transcription kit (transgenes, AT311). Subsequently, qPCR was performed using One‐step System (Applied Biosystems). Melt curve analysis was carried out at the end of the amplification process. The sample was normalized against GAPDH and quantified using the 2^−ΔΔCT^ method. The primer sequences were as follows: *Gapdh* was amplified with F 5′‐ ATGACTCTACCCACGGCAAG ‐3′ and R 5′‐ CTGGAAGATGGTGATGGGTT ‐3′. *Piezo1* was amplified with primers for F 5′‐ GCCGGCTACGGGATTGTG ‐3′ and R 5′‐ GTCCTGGCACAGCTTGAGG ‐3′. *CaMKIIα* was amplified with primers for F 5′‐ TTTGAAAACCTGTGGTCCCGGAACAG ‐3′ and R 5′‐ CCACCCGCATCCAGGTACTG ‐3′. *Fak* was amplified with primers for F 5′‐ GCCACACTGAGCCACTGGC ‐3′ and R 5′‐ CAGTTGGAGCTGTGAGTGCGG ‐3′.

### Intrathecal injection

For intrathecal injection of AAV viruses, 5‐week‐old rats were anesthetized with isoflurane and shaved to expose the skin around the lumbar region. AAV viruses of scramble shRNA and Piezo1 shRNA were diluted and injected with 4 × 10^9^ VG (Viral genomes) each time. AAV viruses were then injected into the lumbar region between L4 and L5 of the spinal cord. Following a 3‐week period after injection, a somatosensory test and immunohistochemistry analysis of rat DRGs were conducted.

All animal procedures strictly adhered to the animal use guidelines of Huazhong University of Science and Technology and received the necessary approvals from the relevant animal use committees.

### Sciatic Nerve Injury

For sciatic nerve injury, adult Sprague‐Dawley rats were anesthetized using isoflurane, and a small incision was made to expose the sciatic nerve. The nerve, positioned 10 mm above the bifurcation into the tibial nerve, was subsequently crushed twice for 15 seconds each time using hemostatic forceps, resulting in a 5 mm‐long crush injury. The crush site was marked with a nylon suture. Only the left side underwent the crush injury procedure, while the contralateral side served as the uninjured control.

All animal procedures strictly adhered to the animal use guidelines of Huazhong University of Science and Technology and obtained the required approvals from the relevant animal use committees.

### Immunohistochemistry

The L4/ L5 DRG tissues or the sciatic nerves were isolated and fixed with 4% paraformaldehyde (PFA) for over 24 h at 4 °C. After fixation, the DRGs were dehydrated through a series of ethanol solutions up to 100% and then immersed in paraffin at 65 °C. The embedded tissues using an embedding machine were subsequently sectioned into 4 µm thick sections. They were deparaffinized and rehydrated to prepare the paraffin sections using xylene, gradient alcohol, and distilled water. The tissue sections were heated in a microwave for 8 min to retrieve antigens using a citric acid (pH 6.0) antigen retrieval buffer. The sections were then incubated in 3% hydrogen peroxide to block any endogenous peroxidase activity in darkness for 25 min. After PBS washed the slices were blocked in 3% BSA prior to being incubated with primary antibodies overnight at 4 °C. The slices were then repeatedly washed with PBS and incubated with fluorescently tagged secondary antibodies for 50 min at room temperature. Finally, slices were washed and counterstained with DAPI for 15 min.

### Behavioral Test

For the evaluation of the sensitivity to mechanical stimulation, the up‐down method was employed to determine the 50% paw withdrawal threshold. Rats were individually placed in glass chambers on a wire mesh table and habituated for 30 min one or two days prior to the test. In this paradigm, testing was conducted by applying a set of von Frey hair filaments and beginning with a 2.0 g filament. The filament was applied to the plantar surface of the hind paw at an angle of 90° through the mesh floor for 10–15 seconds. Stimuli were conducted for at least 2 min. If no paw withdrawal response, a stronger stimulus was applied. Conversely, if a response occurred, a weaker stimulus was chosen for the following trials. Recording began after the first crossed response threshold and continued for an additional four stimuli. The 50% paw withdrawal threshold (g) was calculated based on the responses to this series of stimuli using the Von Frey filament.^[^ ^68^ ^]^

For evaluating the sensitivity to thermal stimulation, the Hargreaves apparatus was employed. Rats were individually placed onto a plexiglass surface and habituated for 30 min one or two days prior to the test. To avoid tissue damage, a cutoff time of 30 seconds was established. The heat source was applied to the plantar surface of the hind paw until the animal withdrew from the noxious thermal stimulus, and the reaction time was recorded. Stimuli were conducted at intervals of at least 10 min. Measurements were repeated three times for each paw.

### Fabrication of PDMS Hydrogel

PDMS hydrogels were prepared using Dow Corning Sylgard 527 A&B silicone dielectric gel (Ellsworth) as previously described.^[^ ^69^ ^]^ By adjusting the ratio of part A to part B components in the mixture, PDMS gels with varying stiffness levels were prepared. For 2 kPa gel, the ratio of A:B was 1.2, for 50 kPa gel the ratio of A:B was 0.3, for 100 kPa gel the ratio of A:B was 0.15, and for 200 kPa gel the ratio of A:B was 0.075. Plates were coated with the hydrogel and incubated for over 8 h at 60 °C. Then the gels were coated with 0.1 mg mL^−1^ poly‐D‐lysine (Sigma) and incubated at 4 °C overnight.

### Quantification and Statistical Analysis

The data presented in all figures are expressed as mean ± S.E.M. For those data that follow a normal distribution, statistical significance was assessed using a two‐tailed Student's t‐test or one‐way ANOVA with GraphPad Prism Software. The data points within each column were derived from multiple independent repetitions of the experiments. Statistical significance was represented as: *p < 0.05, **p < 0.01, ***p < 0.001, ****p < 0.0001.

## Conflict of Interest

The authors declare no conflict of interest.

## Author Contributions

M.L. and W.W. contributed equally to the work. C.M. conceived the experiments and supervised the study. M.L. and W.W. performed most of the experiments and data analysis. J.G. and X.Y. assisted with Western blot and behavioral experiments. H.Z., Z.W., and H.C. assisted with cell biology experiments. S.W. and L.Z. assisted with data analysis. M.L., W.W., and C.M. wrote the manuscript.

## Supporting information



Supporting Information

## Data Availability

The data that support the findings of this study are available from the corresponding author upon reasonable request.
