# Supplementary material for: Piezo1 Regulates Stiffness‐Dependent DRG Axon Regeneration via Modifying Cytoskeletal Dynamics
Source: Adv Sci (Weinh). 2024 Nov 8;11(47):2405705. doi: 10.1002/advs.202405705 (PMC11653623; doi:10.1002/advs.202405705)
Supplement: Supplementary file 1 — Supporting Information [file ADVS-11-2405705-s001.docx]

**Supplementary figures and figure legends**


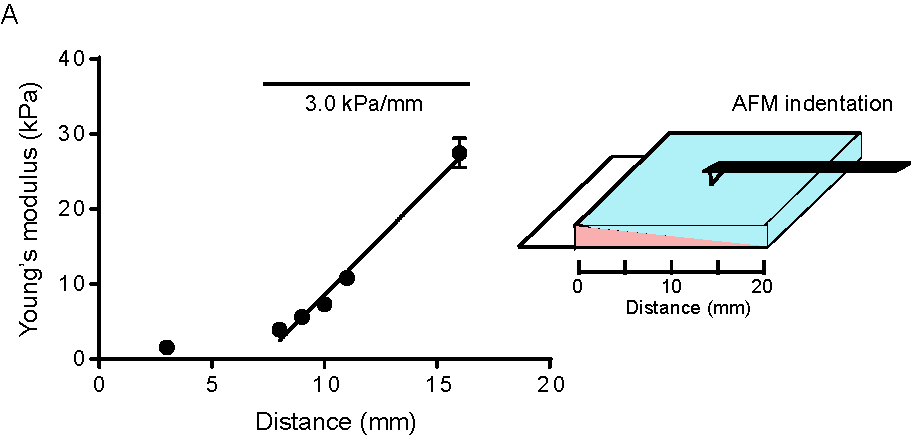


**Supplementary figure 1. The stiffness of gradient PA gels is measured by AFM.**

1. AFM was used to detect the stiffness of gradient PA gels (right) and stiffness gradient of PA gels was quantified as 3.0 kPa/mm (left). The equation of stiffness gradient: y = 3.050x - 22.9.


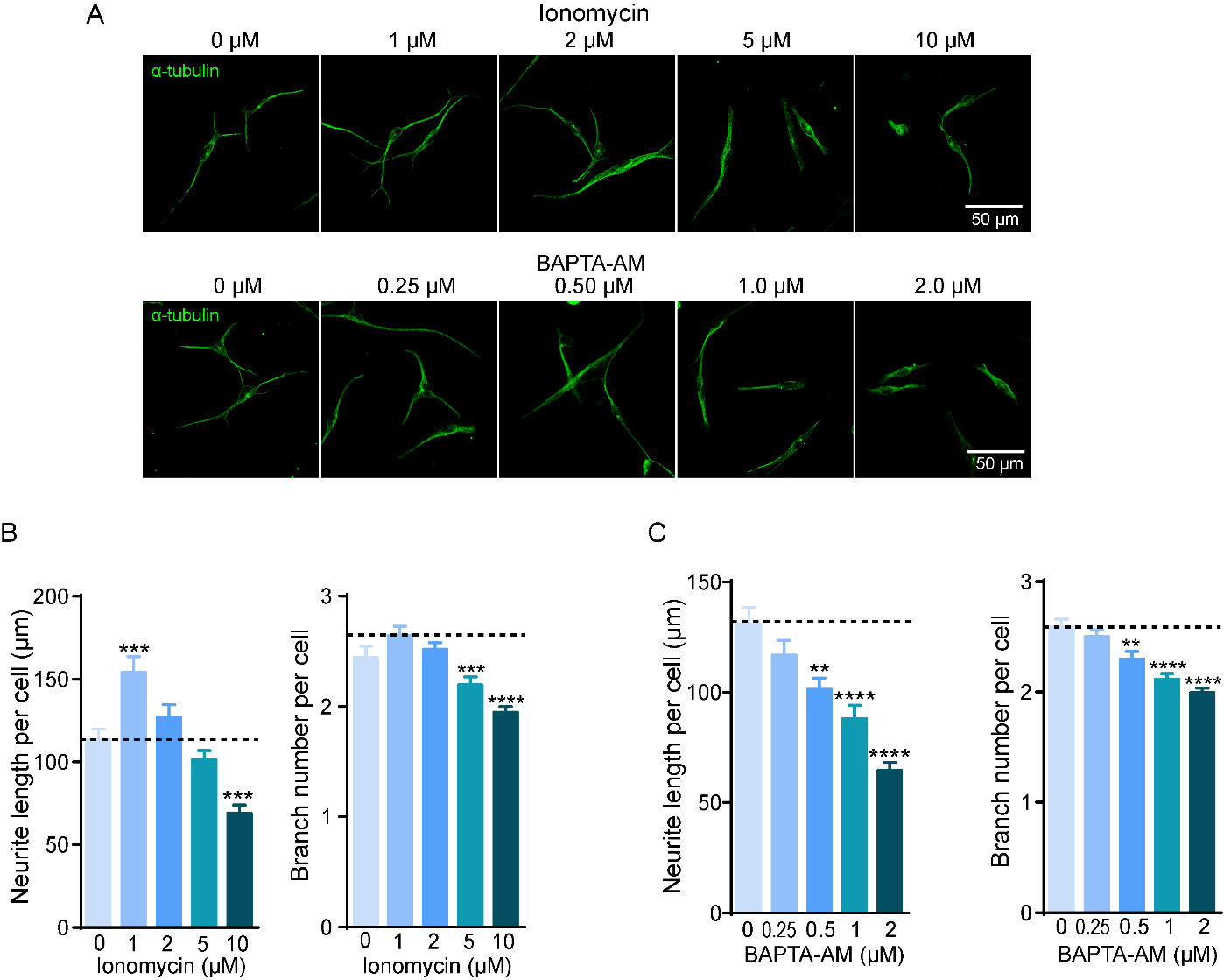


**Supplementary figure 2. Ca^2+^ signaling modulates DRG axon regrowth in a bidirectional regulatory manner.**

1. Representative images of DRG neurons cultured on glass slides and treated with different concentrations of Ionomycin or BAPTA-AM for 24 hours.
2. Quantification of neurite length per neuron as ascribed in panel (A).
3. Quantification of branch number per neuron as ascribed in panel (A).

Error bars denote mean ± SEM; **, p < 0.01; ***, p < 0.001; ****, p < 0.0001, as determined by one-way ANOVA.


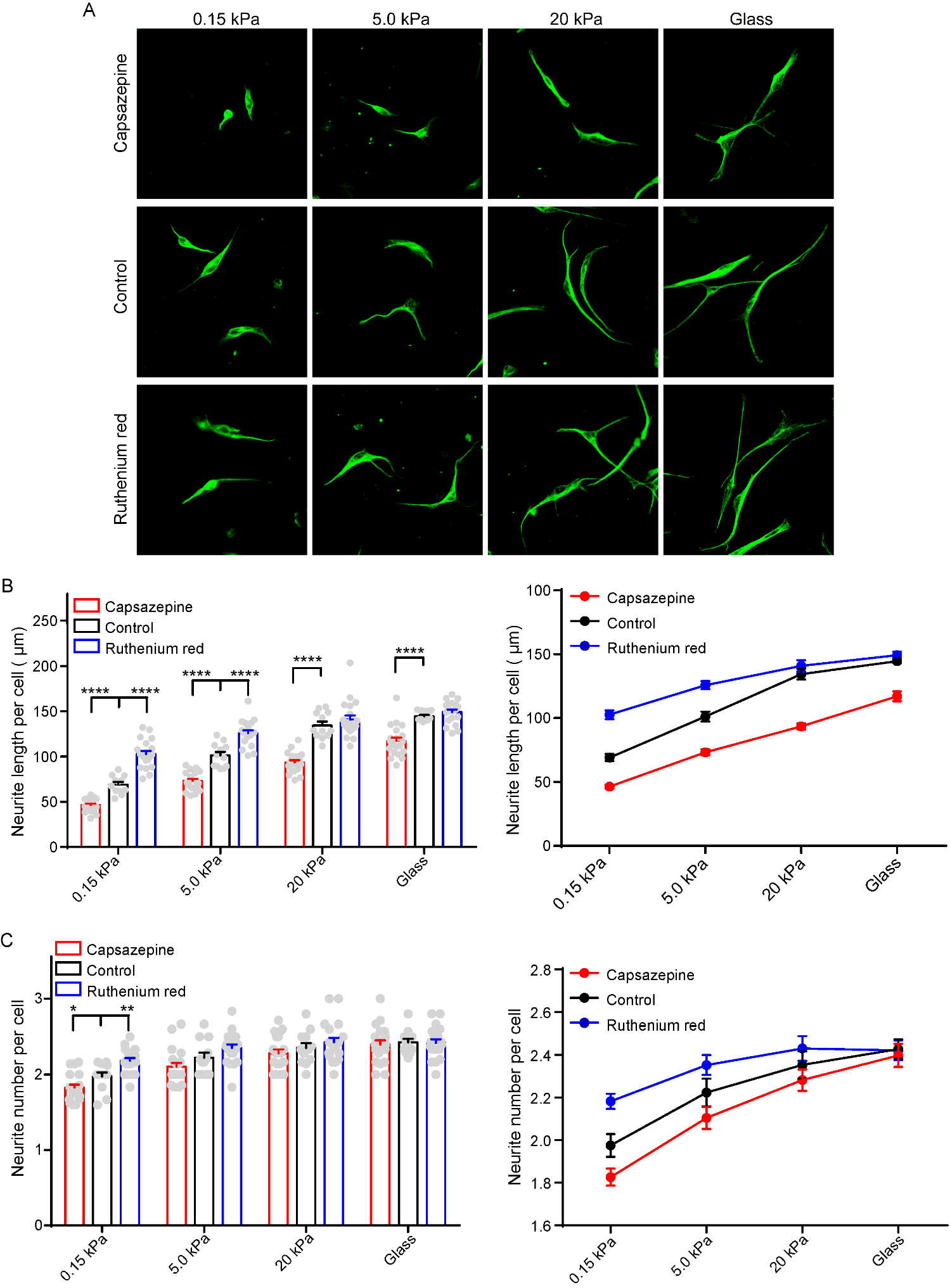


**Supplementary figure 3. Ruthenium red and capsazepine modulate DRG axon regrowth.**

1. Representative images of DRG neurons cultured on 0.15 kPa, 5.0 kPa, 20 kPa PA gels and glass slides and treated with 20 μM Ruthenium red or 2.5 μM capsazepine for 24 hours.
2. Quantification of neurite length per neuron using histograms and line graphs as ascribed in panel (A).
3. Quantification of branch number per neuron using histograms and line graphs as ascribed in panel (A).

Error bars denote mean ± SEM; *, p < 0.05; **, p < 0.01; ****, p < 0.0001, as determined by two-tailed Student’s t-test.


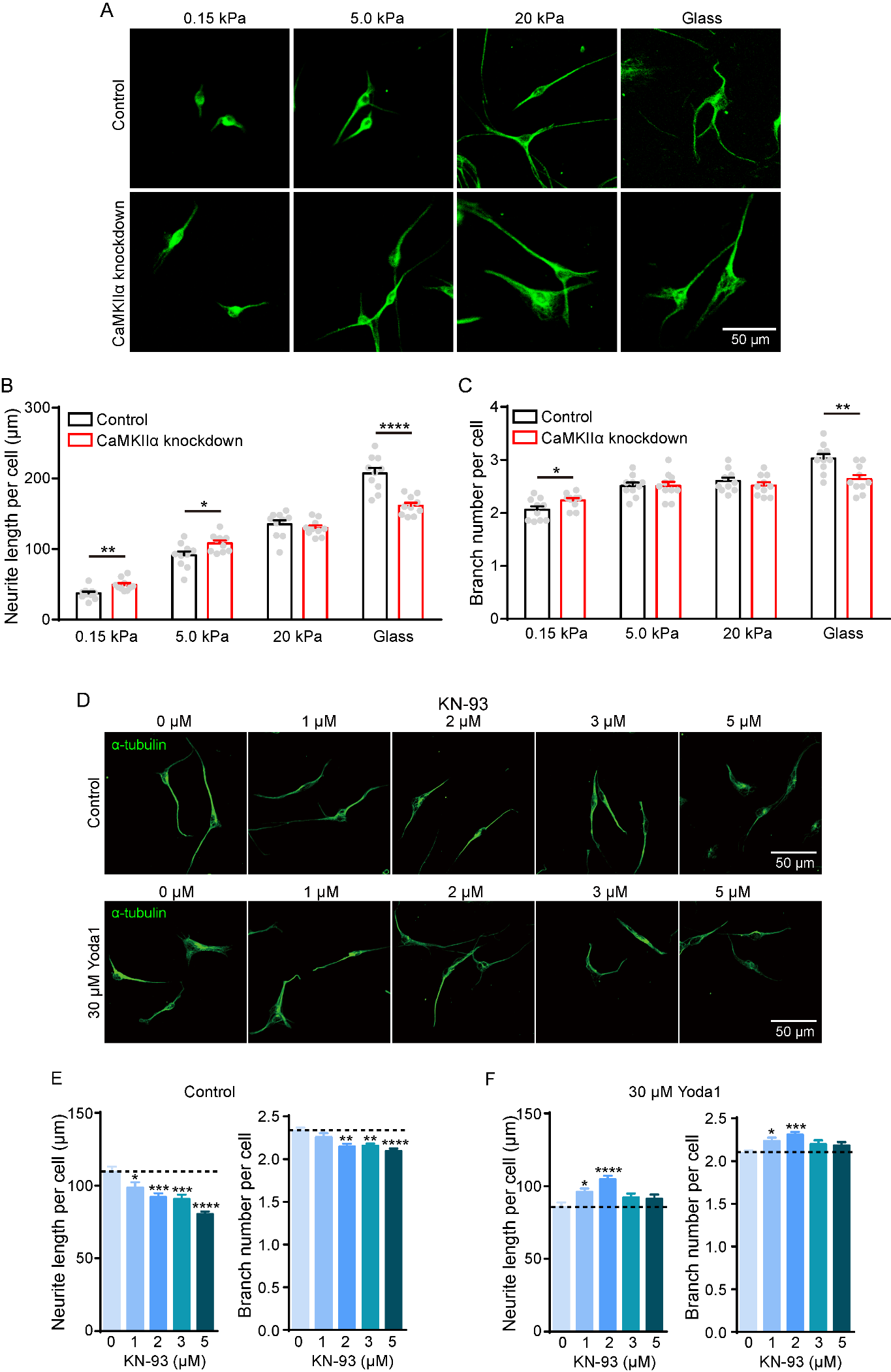


**Supplementary figure 4. CaMKII modulates DRG axon regrowth mediated by stiffness in a bidirectional regulatory manner.**

1. Representative images of DRG neurons infected with control (Scramble shRNA) or CaMKIIα knockdown (CaMKIIα shRNA) virus and cultured on different stiffness substrates for 24 hours.
2. Quantification of neurite length per neuron as ascribed in panel (A).
3. Quantification of branch number per neuron as ascribed in panel (A).
4. Representative images of DRG neurons cultured on glass slides and treated with different concentrations of KN-93 (with or without 30 μM Yoda1) for 24 hours.
5. Quantification of neurite length per neuron as ascribed in panel (D).
6. Quantification of branch number per neuron as ascribed in panel (D).

Error bars denote mean ± SEM; *, p < 0.05; **, p < 0.01; ***, p < 0.001; ****, p < 0.0001, as determined by two-tailed Student’s t-test (B and C) or one-way ANOVA (E and F).


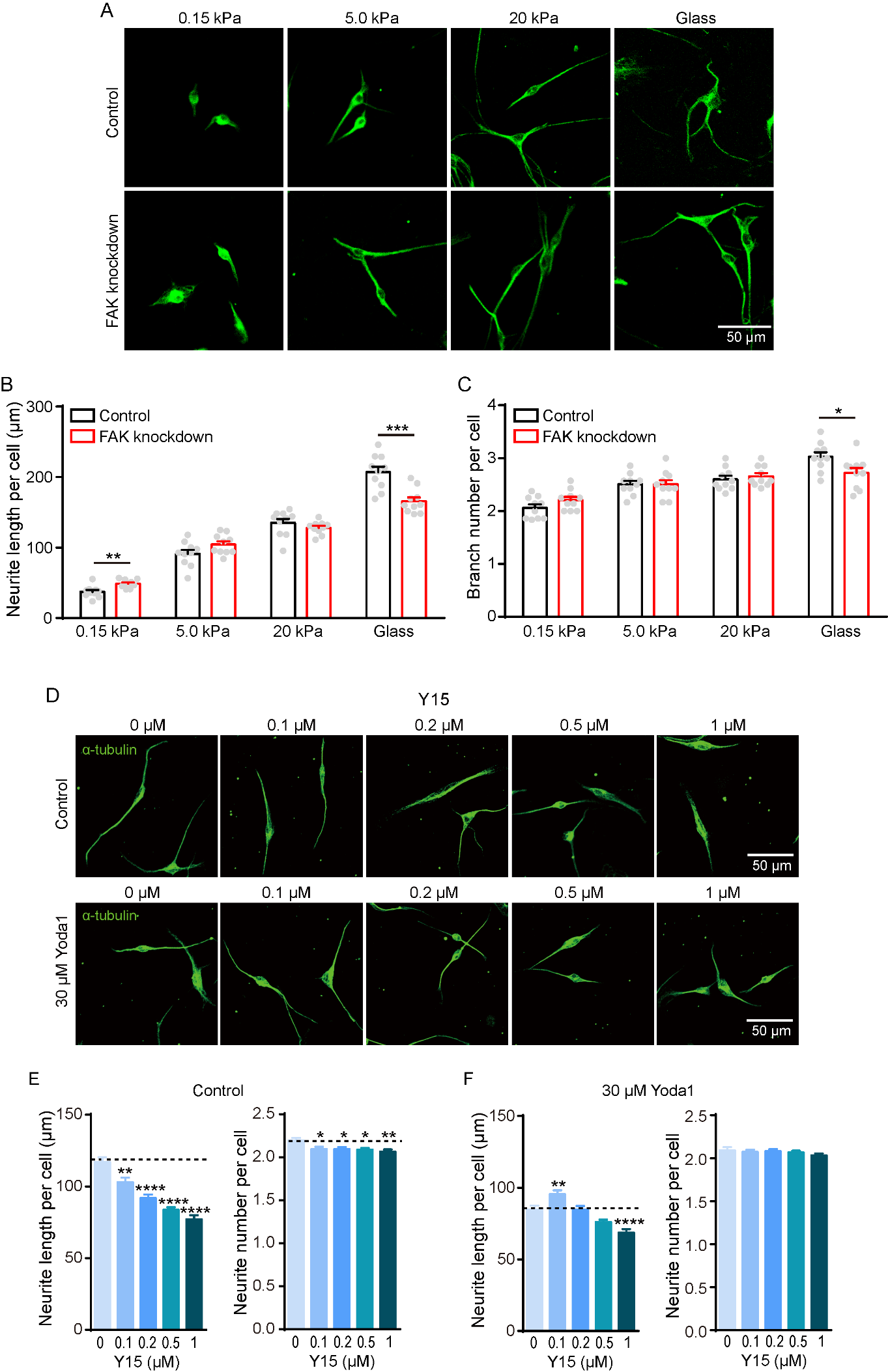


**Supplementary figure 5. FAK modulates DRG axon regrowth mediated by stiffness in a bidirectional regulatory manner.**

(A) Representative images of DRG neurons infected with control (Scramble shRNA) or FAK knockdown (FAK shRNA) virus and cultured on different stiffness substrates for 24 hours.

(B) Quantification of neurite length per neuron as ascribed in panel (A).

(C) Quantification of branch number per neuron as ascribed in panel (A).

(D) Representative images of DRG neurons cultured on glass slides and treated with different concentrations of Y15 (with or without 30 μM Yoda1) for 24 hours.

(E) Quantification of neurite length per neuron as ascribed in panel (D).

(F) Quantification of branch number per neuron as ascribed in panel (D).

Error bars denote mean ± SEM; *, p < 0.05; **, p < 0.01; ***, p < 0.001; ****, p < 0.0001, as determined by two-tailed Student’s t-test (B and C) or one-way ANOVA (E and F).


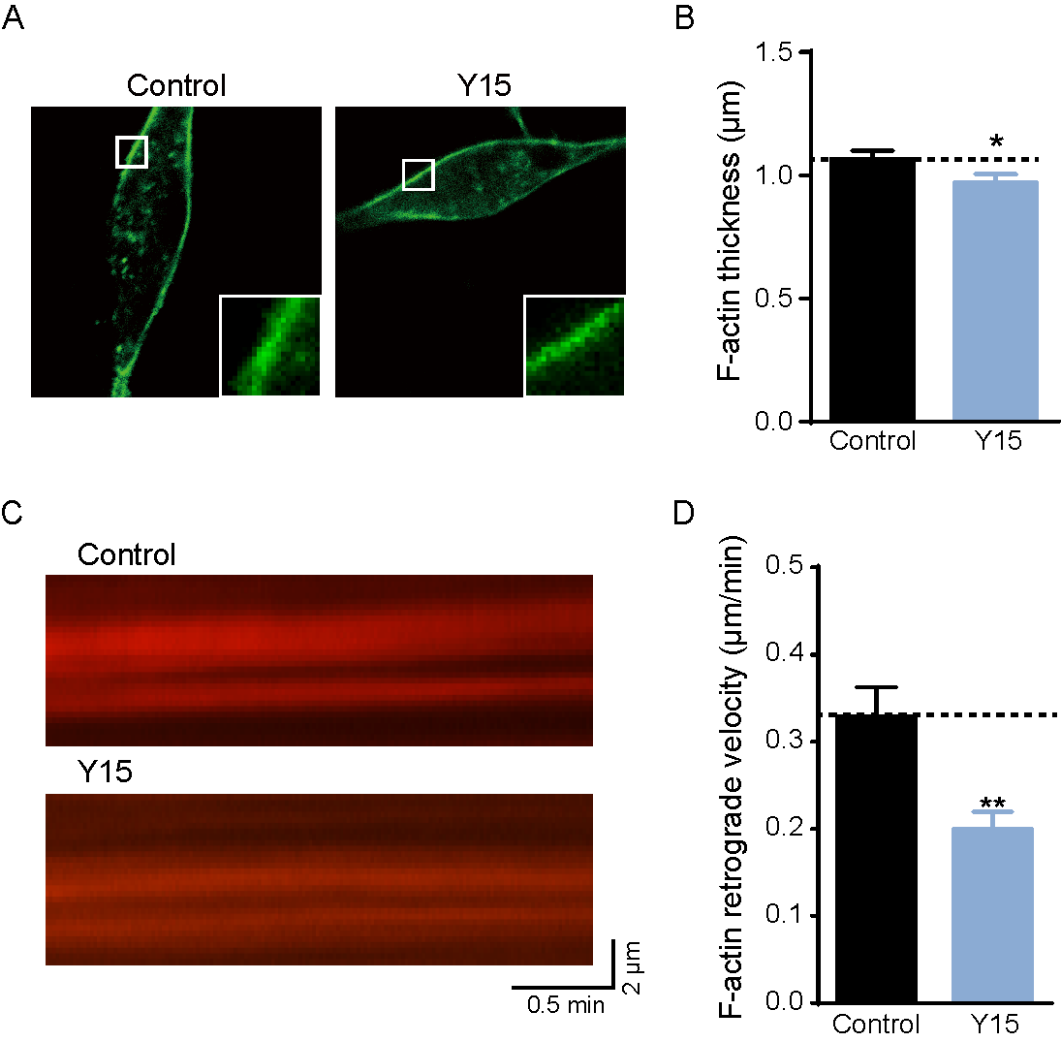


**Supplementary figure 6. FAK modulates the dynamics of actin.**

1. Representative images of F-actin in DRG neurons cultured on glass slides and treated with media (control) or 1 μM Y15 for 24 hours. Phalloidin staining was used to visualize F-actin.
2. Quantification of membrane-located F-actin intensity as ascribed in panel (A).
3. Representative images of F-actin retrograde flow at growth cone of DRG cultured on glass slides and treated with media (control) or 1 μM FAK before recording.
4. Quantification of F-actin retrograde flow velocity as ascribed in panel (C).

Error bars denote mean ± SEM; *, p < 0.05; **, p < 0.01, as determined by two-tailed Student’s t-test.


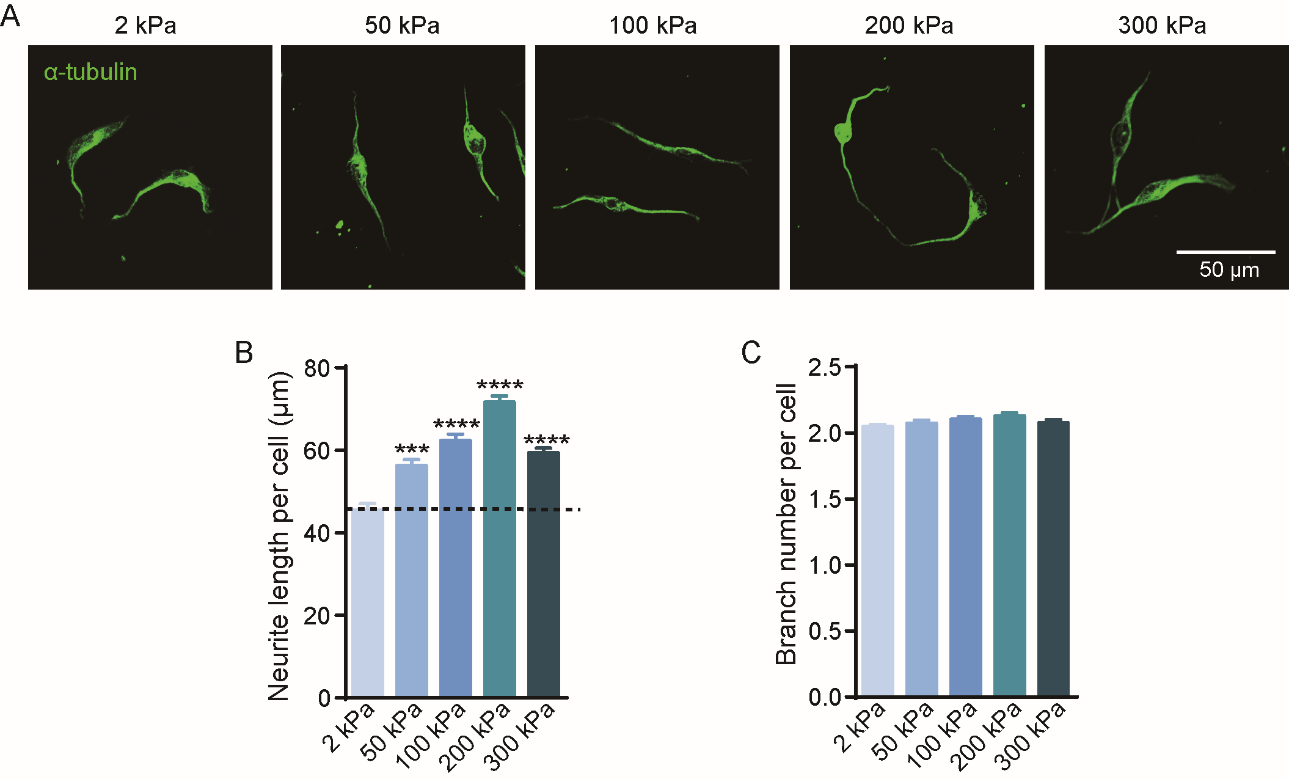


**Supplementary figure 7. Substrate stiffness modulates DRG axon regrowth in a bidirectional regulatory manner.**

1. Representative images of DRG neurons cultured on 2 kPa, 50 kPa, 100 kPa, 200 kPa and 300 kPa PDMS hydrogel for 24 hours.
2. Quantification of neurite length and branch number per neuron cultured on different substrate stiffness as ascribed in panel (A).

Error bars denote mean ± SEM; ***, p < 0.001; ****, p < 0.0001, as determined by one-way ANOVA.


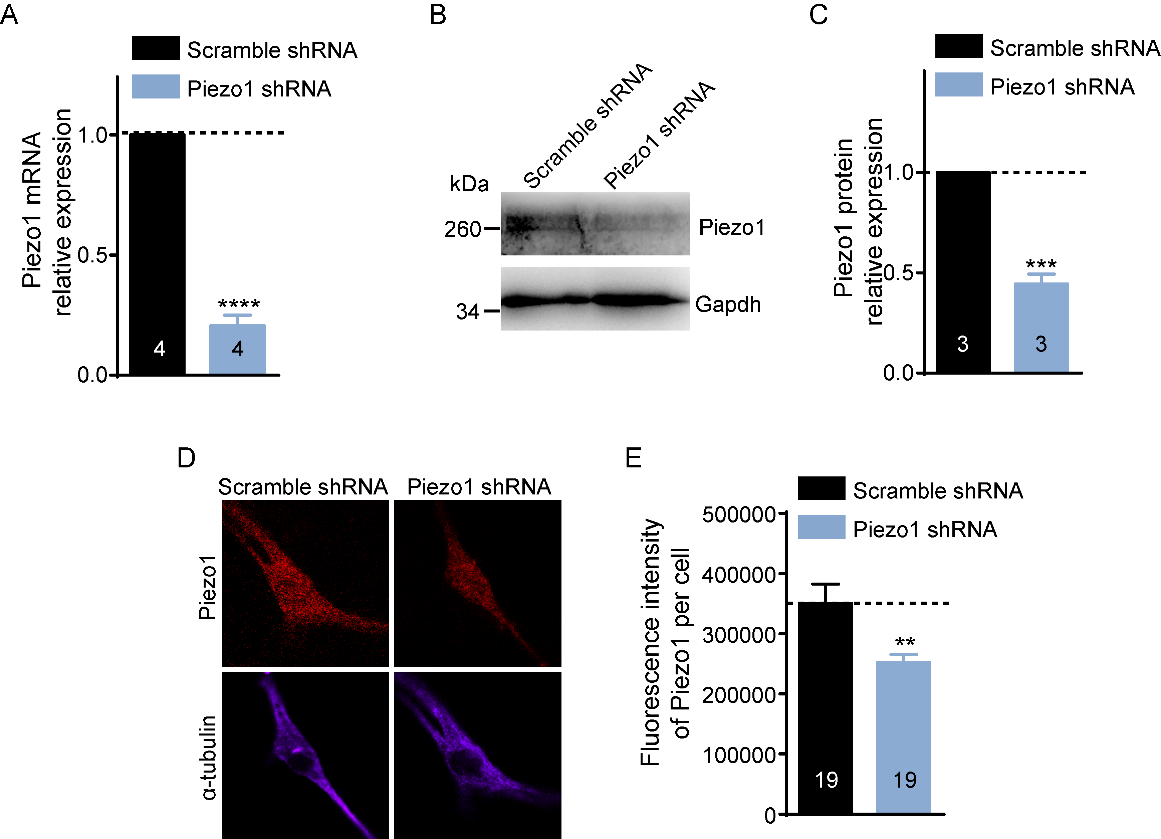


**Supplementary figure 8. Piezo1 is silenced by Piezo1 shRNA in DRGs.**

1. The mRNA expression levels of *Piezo1* in L3-L6 DRGs that were infected with scramble shRNA or Piezo1 shRNA virus was quantified by RT-PCR.
2. Western blots of Piezo1 in whole cell lysis of DRG neurons seeded on glass slides and infected with scramble shRNA or Piezo1 shRNA virus for 6~7 days, GAPDH used as the reference protein.
3. Quantification of Piezo1 in whole cell lysis as ascribed in panel (B).
4. Representative immunofluorescence images of Piezo1 and α-tubulin in DRG neurons infected with scramble shRNA or Piezo1 shRNA virus for 6~7 days and replanted on glass slides for 24 hours.
5. Quantification of Piezo1 fluorescence intensity as ascribed in panel (D), α-tubulin used as the reference protein.

Error bars denote mean ± SEM; **, p < 0.01; ***, p < 0.001; ****, p < 0.0001, as determined by two-tailed Student’s t-test.
